# Differences in temporal processing speeds between the right and left auditory cortex reflect the strength of recurrent synaptic connectivity

**DOI:** 10.1371/journal.pbio.3001803

**Published:** 2022-10-21

**Authors:** Demetrios Neophytou, Diego M. Arribas, Tushar Arora, Robert B. Levy, Il Memming Park, Hysell V. Oviedo

**Affiliations:** 1 CUNY Graduate Center, New York, New York, United States of America; 2 Department of Neurobiology and Behavior, Stony Brook University, Stony Brook, New York, United States of America; 3 Biomedicine Research Institute of Buenos Aires, Buenos Aires, Argentina; 4 Biology Department, The City College of New York, New York, New York, United States of America; University College London, UNITED KINGDOM

## Abstract

Brain asymmetry in the sensitivity to spectrotemporal modulation is an established functional feature that underlies the perception of speech and music. The left auditory cortex (ACx) is believed to specialize in processing fast temporal components of speech sounds, and the right ACx slower components. However, the circuit features and neural computations behind these lateralized spectrotemporal processes are poorly understood. To answer these mechanistic questions we use mice, an animal model that captures some relevant features of human communication systems. In this study, we screened for circuit features that could subserve temporal integration differences between the left and right ACx. We mapped excitatory input to principal neurons in all cortical layers and found significantly stronger recurrent connections in the superficial layers of the right ACx compared to the left. We hypothesized that the underlying recurrent neural dynamics would exhibit differential characteristic timescales corresponding to their hemispheric specialization. To investigate, we recorded spike trains from awake mice and estimated the network time constants using a statistical method to combine evidence from multiple weak signal-to-noise ratio neurons. We found longer temporal integration windows in the superficial layers of the right ACx compared to the left as predicted by stronger recurrent excitation. Our study shows substantial evidence linking stronger recurrent synaptic connections to longer network timescales. These findings support speech processing theories that purport asymmetry in temporal integration is a crucial feature of lateralization in auditory processing.

## Abstract 2

This study uses the mouse as a model system that captures relevant features of human lateralized processing of speech and music, revealing a novel mechanism whereby stronger recurrent activity in the Right Auditory Cortex led to longer temporal integration compared to the Left (consistent with the temporal scales of syllabic and supra-syllabic processing).

## Introduction

Social communication calls have myriads of constituent sounds that are temporally and spectrally dynamic. The auditory system must have processes in place to quickly decode and encode ethologically relevant features of an auditory signal to elicit an appropriate response. Hemispheric asymmetry in sound processing (i.e., lateralization) has long been proposed to be critical in the dynamic processing of speech sounds. Human studies have shown that the left superior temporal gyrus (STG) is more capable of integrating information over a shorter timescale and plays a greater role in speech perception and phonological processing than the right [1–3]. On the other hand, the right STG has longer integration windows to potentially subserve the processing of suprasegmental information [4,5]. The unanswered question remains: What neural mechanisms underlie these differences in temporal integration? One possible mechanism we dissect in this study is interhemispheric differences in excitatory recurrent connections. This would introduce positive feedback that would counteract the exponential decay of individual neurons within a neural population, leading to effectively slower temporal dynamics with a longer time constant.

Studies of animal models can provide more mechanistic insight regarding circuit function. Circuit mapping of the mouse auditory cortex (ACx) has shown that the synaptic organization of Layer 3 (L3) differs between the 2 hemispheres. In the left ACx, principal neurons in L3 receive out-of-column excitatory input from L6 cells located in higher frequency bands, whereas in the right ACx, the same pathway has balanced frequency projections across the tonotopic axis. This lateralized synaptic organization is in turn associated with differences in sound-evoked activity in L3 [6]. Behavioral studies in gerbils have reported asymmetries in temporal processing. Lesions in the right ACx impact discrimination of frequency sweep direction, suggesting it plays a role in processing global temporal cues. Whereas lesions in the left ACx impact discrimination of gap durations, implying a role in processing local temporal cues [7].

Here, we tested directly whether there are differences in the temporal integration properties between the auditory cortices and dissected the underlying circuit dynamics. To assess cortical circuit mechanisms that could underlie hemispheric asymmetry in temporal processing, we used circuit-mapping techniques to screen for connectivity differences in excitatory pathways. We show novel differences in recurrent connectivity between the hemispheres, particularly in the superficial layers. To examine how these asymmetries in recurrence translate into differences in temporal integration, we recorded spontaneous spikes from awake mice and developed a statistical method to estimate time constants and their uncertainty from spike trains. Our method utilizes the dichotomized Gaussian (DG) spiking model to generate surrogate spike trains with a data matching autocorrelation function. The estimated time constants from each neuron recorded in superficial layers of the left and right ACx were aggregated to form a distribution over the network time constants. We found significant differences in temporal integration consistent with the timescale of hypothesized lateralized auditory signal processing. Numerous models of neural architectures have been proposed to account for observed differences in integration timescales throughout the brain. Here, we show, for the first time, compelling evidence of differences in synaptic circuit organization that translate into distinct temporal integration windows.

## Results

### Lateralized connectivity motifs are found in every layer of the auditory cortices

**Fig 1:**
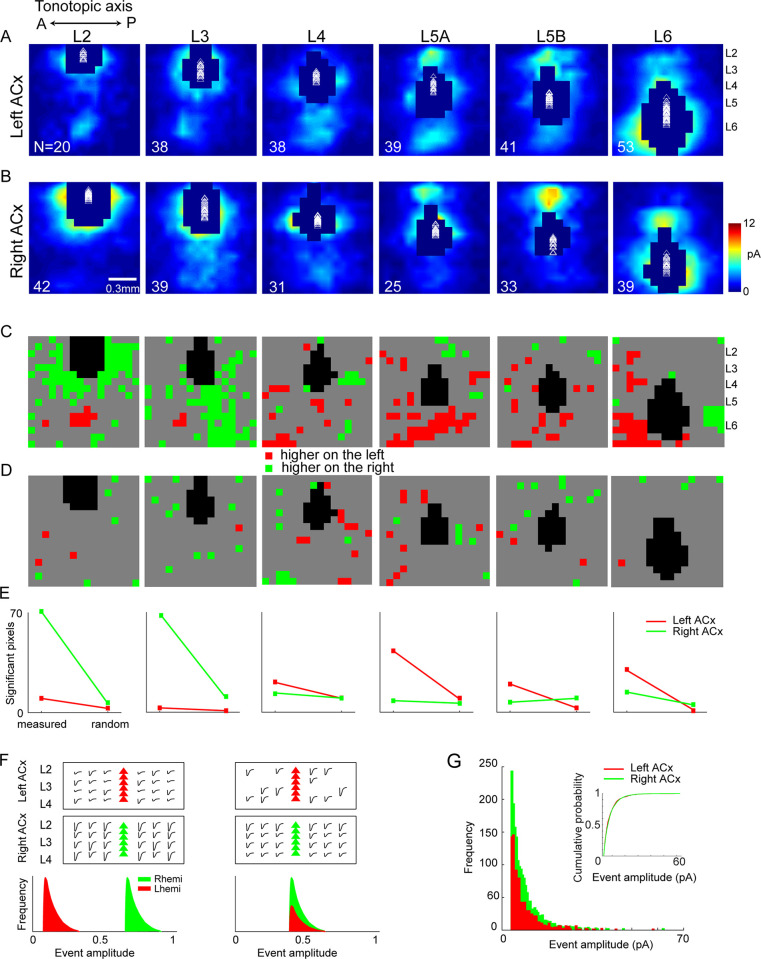
Summary of excitatory pathways in the left and right ACx across all cortical layers. (A, B) Patterns of excitatory synaptic input were recorded using LSPS as described in the text. Maps were averaged over all cells in each layer and then interpolated for clarity. Masked areas indicate direct hits in >50% of cells for that region. White triangles denote location of somata. Laminar boundaries were defined with respect to the fractional distance from the L1/L2 boundary to the white matter. L5A and L5B were defined as the upper and lower 50% of L5, respectively. N indicates number of recorded cells for each panel. (C) Pixel-wise significance maps (*p* < 0.05, unpaired 2-tailed *t* tests) for response amplitude in left vs. right ACx. *Red* and *green* denote significantly higher average response in the left and right ACx, respectively. *Gray* denotes no significant difference. (D) Same as C but maps from both hemispheres were pooled and assigned to 2 groups at random with the same total N shown in A, B. (E) Graphs of the significant pixel counts for measured (C) and random (D) comparisons. (F) Models depicting potential synaptic mechanisms underlying the right ACx’s higher excitatory connectivity in superficial layers compared to the left ACx. The left panel shows both cortices with a similar pool of presynaptic sources of EPSCs (traces) projecting onto postsynaptic targets (triangles), but the distribution of event amplitudes differs. The right panel shows the left and right ACx differ in their pool of presynaptic inputs but have a similar event amplitude distribution. (G) Observed frequency of event amplitudes in superficial layers of the left and right ACx. Inset shows empirical cumulative distribution of event amplitudes. The data underlying all the plots in this figure are included in S1 Data. ACx, auditory cortex; LSPS, laser scanning photostimulation.

To screen for hemispheric differences in the organization of excitatory pathways, we used glutamate uncaging-based laser scanning photostimulation (LSPS; [8]). We performed voltage-clamp recordings on principal neurons in Layers 2 to 6 of the left (*n* = 229) and right (*n* = 209) ACx, (Fig 1A and 1B). The uncaging stimulus grid covered the entire primary ACx and all cortical layers (total area of 1.125*1.125 mm, 256 stimulation sites/map pixels). Hence, we specifically measured only intracortical sources of synaptic input. We focused on excitatory input by holding the membrane potential at the reversal for inhibition (-70 mV). To assess potential synaptic connectivity differences in each cortical layer, we performed a statistical comparison of the population data underlying corresponding map pixels from each hemisphere. We found statistically significant differences in the strength and organization of synaptic input between most layers of the auditory cortices (Fig 1C). To assess the likelihood that these differences were by chance, we randomly assigned each pixel to the left or right ACx. We largely found fewer significant differences between the hemispheres when the pixels were randomized (Fig 1D).

A large source of presynaptic input to L2 in the right ACx arose from intralaminar and other superficial layers (Fig 1A–1C). In contrast, in the left ACx, the most influential presynaptic pathway to L2 arose from deep layers (L5/6), as was reported previously [9]. There were also hemispheric differences in the organization of input to L3, which were also previously described in detail [6]. Intracortical input to L4 was stronger in the left ACx compared to the right (Fig 1A–1C). Layers 5A and 5B are functionally distinct in the ACx [10,11], and the organization of their intracortical synaptic input appears to be lateralized. In the left ACx, there was greater ascending input to L5A compared to the right ACx. Conversely, there was greater descending input to L5B in the right ACx compared to the left ACx (Fig 1A–1C). Finally, L6 had complementary patterns of intralaminar synaptic input along the tonotopic axis: The left ACx had greater input arising from higher frequency bands, and the right ACx from lower frequency bands (Fig 1A–1C). A comparison of the number of measured and random pixels for each layer revealed that the most abundant differences in synaptic input between the hemispheres arose in the superficial layers of the left and right ACx (Fig 1E). Several synaptic mechanisms could underlie the observation of more significant hotspots in superficial layers of the right ACx compared to the left. One possibility is that each hemisphere has a similar pool of presynaptic inputs in these layers, but individual synaptic events were larger in the right ACx compared to the left (Fig 1F left). Another possibility is that the distribution of the synaptic events’ amplitudes is similar between the hemispheres, but there is a larger pool of presynaptic inputs in the right ACx compared to the left (Fig 1F right). To disentangle these possibilities, we analyzed the distribution of event amplitudes in superficial layers (L2-4). We randomly chose the same number of cells to analyze from each hemisphere (*n* = 96), and the threshold for synaptic events was set to above the average baseline events. Fewer synaptic events met the threshold criteria in the left ACx (*n* = 1,090) compared to the right (*n* = 1,761, Fig 1G). Additionally, the distribution of event amplitudes was similar between the auditory cortices (Fig 1G inset). This supports the prediction that there is a larger pool of presynaptic inputs in superficial layers of the right ACx.

### Lateralized recurrent connectivity in superficial layers of the auditory cortices

**Fig 2:**
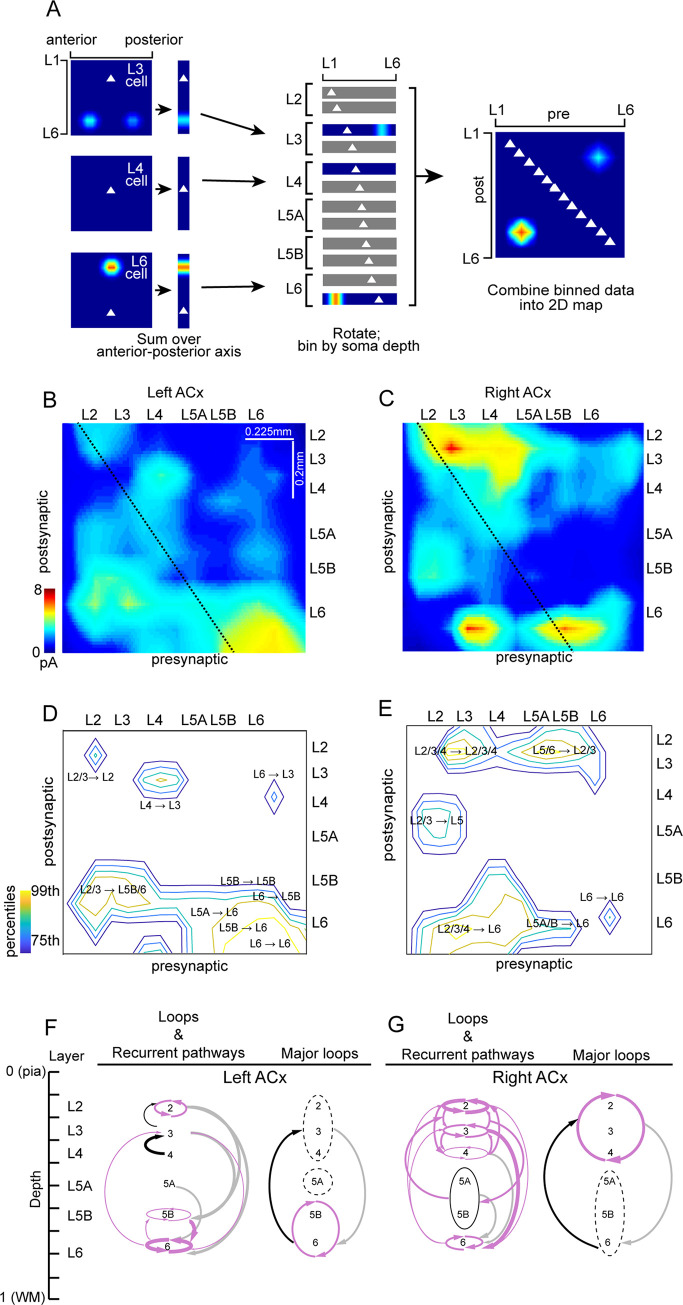
Recurrent connections are significantly stronger in superficial layers of the right ACx. (A) Schematic diagram of the generation of laminar input–output maps [14]. *Left*, 2D LSPS-derived input maps for each cell were summed over the anterior-posterior axis to produce 1D maps of input strength vs. cortical depth (vertical strips). Note that anteroposterior information is discarded, such that the 2 L6 input hotspots for the L3 cell (top) collapse to a single spot. Triangles denote soma location. *Middle*, the 1D maps were rotated 90 degrees (for graphic clarity), sorted by cortical depth of the soma, and binned (bin size = 80 μm). *Right*, the binned maps were combined into a single 2D map of presynaptic input location (x axis) vs. binned postsynaptic soma location (y axis). Maps were interpolated for display. (B) Input–output map for the left ACx, constructed as shown in A. Diagonal line indicates x = y with respect to cortical depth. The diagonal does not span the full x axis because recorded cell bodies (y axis) were confined between L2 and L6, whereas the stimulation grid (x axis) extended more broadly from L1 into the white matter. (C) Same as B but for the right ACx. (D) Same map as in B but showing only pathways in the 75th percentile and above in the left ACx. (E) Same as D but for the right ACx. (F) Summary of loops and pathways in the 75th percentile and above in the left ACx and right ACx (G). In F and G, the arrow thickness indicates strength of the pathway (thickest in the 99th percentile to thinnest in the 75th percentile), ascending pathways are shown in black, descending in gray, recurrent in violet, and open loop in dashed line. The data underlying all the plots in this figure are included in S1 Data. ACx, auditory cortex; LSPS, laser scanning photostimulation.

Neural circuits composed of neurons with short time constants can effectively have long temporal memory and computation by forming a long feedforward chain or concise recurrent feedback loops [12]. Therefore, we investigated whether the synaptic connectivity differences translate into systematic differences in the recurrent interlaminar feedback. To compare the relative strength of interlaminar pathways between the hemispheres, we computed connectivity matrices for the left and right ACx. These input–output matrices (presynaptic-postsynaptic) summarize the organization of local excitatory networks [13,14]. We ordered maps according to the cortical depth of the soma and summed the 2D LSPS-derived input maps for each cell over the tonotopic axis (across the horizontal dimension), which produced vectors of input strength as a function of cortical depth (Fig 2A). Therefore, each neuron’s input vector represents presynaptic input from different laminar locations. Each row in the laminar connectivity matrix represents input to that specific laminar location, and each column represents synaptic output from that laminar location. Very local connections (<50 μm) within each layer lie along the diagonal and were under-sampled due to direct excitation [13]. In a previous study, we determined that neuronal density and photoexcitability of individual cells did not significantly differ between the hemispheres; therefore, we did not normalize maps by these factors [6].

The most striking asymmetry in the laminar connectivity matrices was the stronger synaptic connections in superficial layers of the right ACx compared to the left (Fig 2B and 2C). To quantify the strengths of the pathways, we calculated fractional input and output. In this analysis, we summed along rows and columns and normalized by the total [14]. The fractional input and output of Layers 2 to 4 was significantly greater in the right ACx (*p* = 0.0079 for input, *p* = 0.0411 for output). In the deeper layers, input to L6 was significantly greater in the left ACx (*p*<<0.001), but there was no hemispheric difference in the output. Significant hemispheric differences in intralaminar and interlaminar loops were a major theme in the organization of auditory circuits. Using the input/output laminar connectivity matrices of the left and right ACx, we examined pathways in the 75th percentile to capture the most significant trends. In the left ACx, we observed strong intralaminar recurrent connectivity only in L6 and to a lesser extent in Layers 2 and 5B (Fig 2D). The strongest interlaminar recurrent connections were observed between deep layers, where L5B and 6 form nested loops (intralaminar loops within L5B and 6, which are in turn connected to each other). In contrast to the sparser prevalence of recurrent connections and loops in the left ACx, these were the dominant motifs in the right ACx. All cortical layers (except L5) in the right ACx were part of nested loops: At the innermost level, there were intralaminar loops (input returning to the same layer), followed by local interlaminar loops between neighboring layers, and transcortical loops that coupled superficial and deep layers (Fig 2E and 2G). On the whole, we identified 5 recurrent loops in the left ACx and 10 in the right ACx. Taken together, these widespread loops of recurrent connections suggest the right ACx may have different temporal filtering properties compared to the left ACx.

### Hemispheric differences in recurrent activity

**Fig 3:**
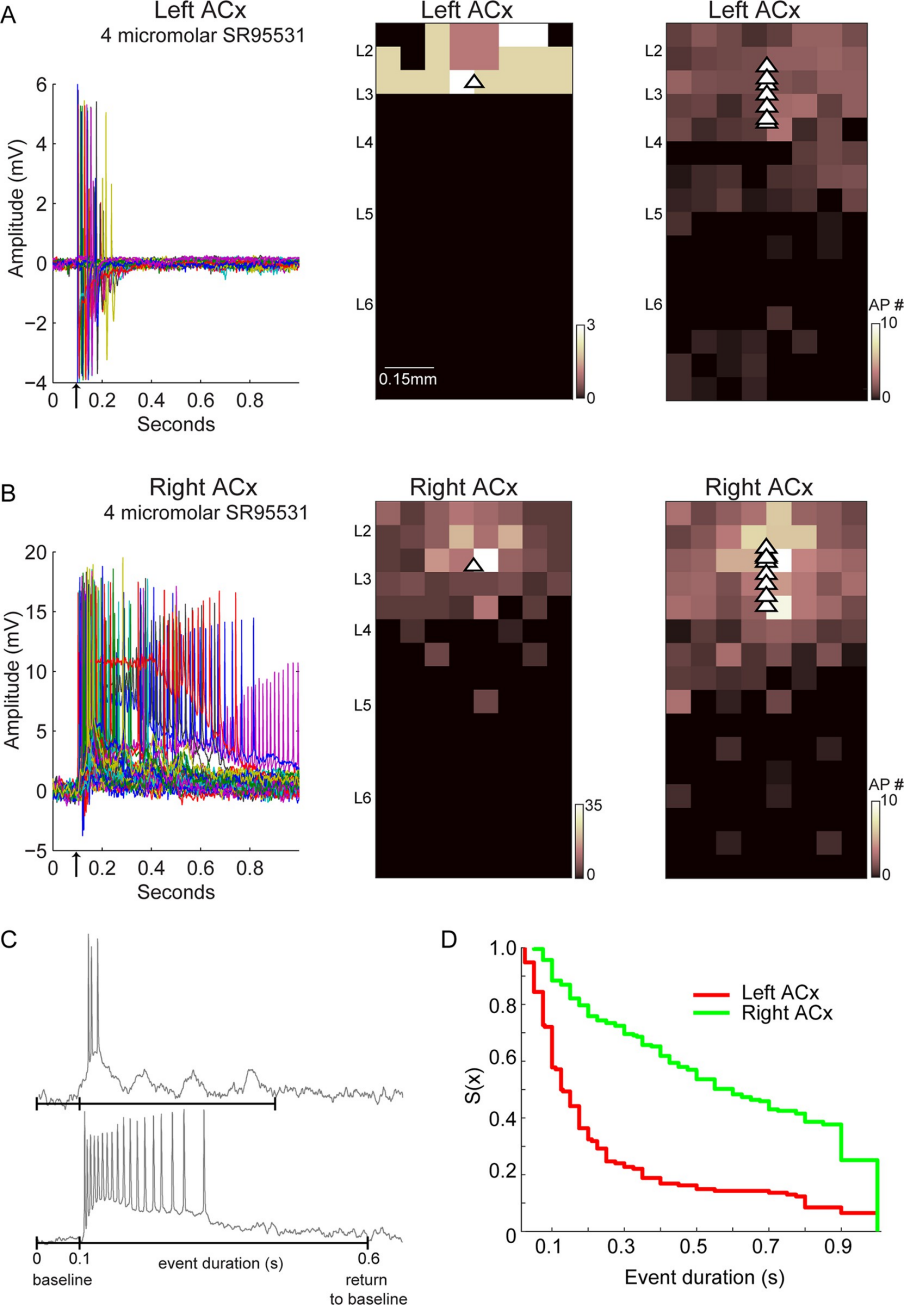
Stronger network events in the right ACx. (A, left) All traces of cell-attached recording from an L3 excitatory neuron in the left ACx with 4 μm concentration of SR95531 in the bath. Black arrow on the x axis marks the onset of photostimulation. (A, middle) Map of action potentials evoked in the cell shown in the left panel and the population of cells recorded in the left ACx (*n* = 8, right). Triangles show position of the cells recorded. (B, left and middle) Same experimental conditions as in A, but conducted in the right ACx of the same animal. (B, right) Population map of all cells recorded in the right ACx (*n* = 8). (C) Duration of events was calculated from the onset of the first action potential to the return of the membrane potential back to baseline period (see Methods for details). (D) The duration of network events is shown using the survival function, which demonstrates that duration times are shorter in the left ACx compared to the right (*n* = 16). All cells were mapped using the same number of stimulus sites (i.e., same stimulus grid). The data underlying all the plots in this figure are included in S1 Data. ACx, auditory cortex.

A potential result of stronger recurrent excitatory connectivity in a circuit is the ability to generate longer network events [15,16]. To test whether the greater recurrent excitatory connectivity observed in the right ACx leads to stronger and longer recurrent activity compared to the left ACx, we used experimental conditions to disinhibit slices [14]. The input–output connectivity maps in Fig 1 were obtained in a slice preparation that reduced excitation to prevent polysynaptic activity [8]. Therefore, to promote excitation in the slice, we partially blocked inhibitory circuits using the GABA_A_ receptor antagonist SR95531 (see Methods for more experimental details). We performed LSPS, recorded in the cell-attached configuration from excitatory neurons in L2-4, and compared network events in the left and right ACx in slices from the same animal. Network events were only triggered by photostimulation and were largely initiated by stimulating sites in superficial layers of the ACx. A representative example of events triggered in the left and right ACx of the same animal using the same concentration of SR95531 shows a greater number of action potentials generated per stimulation site and more sites evoking action potential firing in the right ACx (Fig 3A and 3B left and middle panels). This difference in activity was significant across animals (Fig 3A and 3B right panels, *n* = 16 cells, 4 animals, *p* < 0.001, Wilcoxon rank sum). We also quantified the duration of these network events: the time from the first photostimulation-evoked action potential to the return of the membrane potential back to the pre-stimulus baseline (Fig 3C). The duration of network events was significantly longer in the right ACx compared to the left (Fig 3D, *p*<<0.001 Wilcoxon rank sum.) The long duration and variability of these events suggest that the recurrent excitation results from polysynaptic circuits. In summary, disinhibiting the slice revealed the network-wide impact of stronger recurrent excitatory connectivity in the right ACx.

Hemispheric differences in membrane properties could also contribute to network activity and time constants. To examine this possibility, we compared the current-voltage and current-firing rate relationship of neurons in superficial layers of the left and right ACx (S1 Fig). We found no hemispheric differences between these input–output relationships (I/V, *p* = 0.71 *n* = 10; I/F, *p* = 0.84 *n* = 10). Hemispheric differences in resting membrane potential were also not significant (*p* = 0.86, mean left ACx = −73.4 mV, sem = 1.24, *n* = 28; mean right ACx = −73.2 mV, sem = 1.23, *n* = 28).

### Network time constant is longer in superficial layers of the right auditory cortex

**Fig 4:**
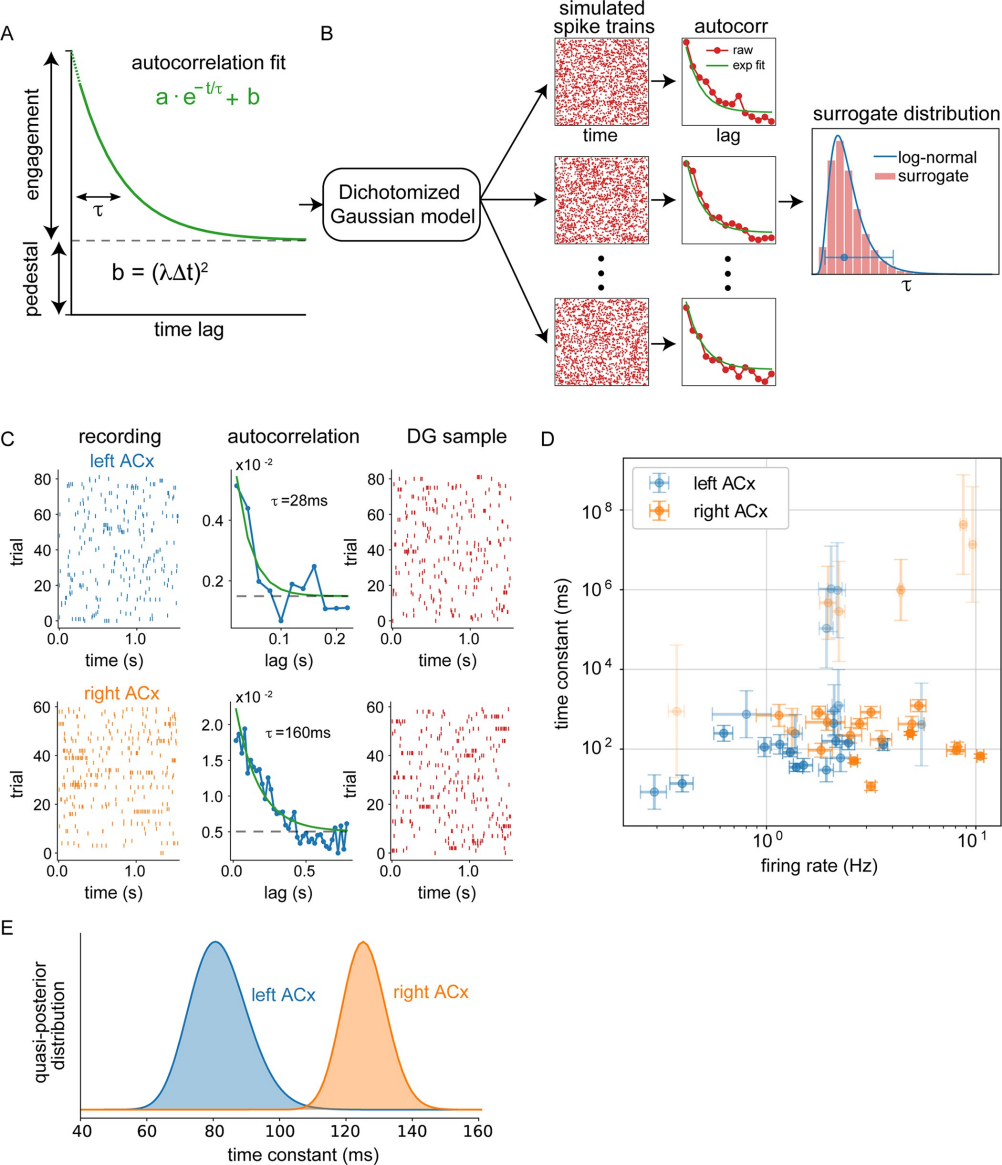
Timescales of neural activity were longer in the right ACx. (A) Schematic of the fitted exponential decay and its parameters. (B) Sketch of the procedure to estimate the bias and uncertainty in the time constant of single neurons. We used the DG model with neuron-specified exponential autocorrelations to generate surrogate data, re-fit exponentials, and extract surrogate time constants. Finally, we fit a lognormal distribution to the surrogate time constants to estimate the bias and variance. (C) Example left and right ACx data, autocorrelations, and sample of surrogate data generated from DG model with the extracted autocorrelation. (D) Bias corrected time constant with its uncertainty against mean firing rate for each neuron. Time constant error bars are the standard deviation of the surrogate lognormal distribution. Firing rate error bars are the standard error over trials. (E) Time constant posterior distributions obtained by integrating the single neuron observations for each hemisphere. The total number of cells in panels D and E is 45 (23 right ACx, 22 left ACx). The data underlying all the plots in this figure are included in S1 Data and S1 File. ACx, auditory cortex; DG, dichotomized Gaussian.

To determine if hemispheric differences in recurrent connectivity impacted the network dynamics, we measured the time constant of the spontaneous neural activity in awake animals. In the first-order approximation of a system, the causal link between recurrent anatomy and time constant is based on the fact that excitatory feedback increases the time constant, therefore it provides an approximation of the dynamics by measuring how quickly the correlation in the neural activity decays over time. Without recurrent connectivity, the time constant of a single neuron’s activity is at the scale of the membrane time constant and delays. Whereas with recurrent connectivity, the fluctuations in the network activity can decay more slowly (i.e., longer time constant) due to effective self-excitation (see Methods for mathematical explanation). Therefore, we studied whether hemispheric differences in recurrent connectivity translate into a difference in the network time constants reflected in a single neuron’s spontaneous activity in the absence of auditory stimuli. We performed cell-attached recordings in superficial layers (150 to 500 μm below the cortical surface) of the left and right ACx. Because anesthesia can reduce the contribution of network activity [17], we conducted experiments in head-fixed awake mice. Neurons in both auditory cortices were active in the absence of auditory stimuli and displayed activity that suggested the presence of temporal correlations (Fig 4C).

We extracted the time constant of individual cells by fitting an exponential decay with a pedestal to their autocorrelograms (Fig 4A). However, precise quantification of time constants from spike trains and their autocorrelograms can be challenging [18,19] especially for neurons with low firing rates or low degrees of engagement with the network. In such a low signal-to-noise-ratio (SNR) regime where spike trains (signal + noise) may not be very informative about the network time constant (signal), the estimated time constant can exhibit large bias and variance. Unfortunately, bootstrapping or resampling procedures cannot practically correct for these issues. For example, if there are only a handful of spikes, bootstrap samples may consistently provide very small time constants such that the variance of the estimator is close to zero. However, in this low firing rate regime, the uncertainty of the estimator should be large if we properly accounted for the spiking noise—resampling low SNR data combined with a strongly biased estimator can create an illusion of a (misleading) high SNR conclusion.

To better estimate the bias and variance of the estimated time constant itself, we used a method for generating spike trains from a parametric surrogate distribution (Fig 4B, Methods). Briefly, for each neuron, we used the exponential fit of the data’s autocorrelation to build a probabilistic model (see Methods) that we used to generate random data that reproduces the given autocorrelation. We generated surrogate data from this model that matched the length and sampling rate of the recording. By construction, the generated data reproduced the firing rate and autocorrelation of the original data on average, while single realizations showed variability determined by the assumed noise. We then re-fit exponential decays to the surrogate data and extracted surrogate time constants from each of the realizations. Using this surrogate time constant distribution, we corrected the bias by assuming that the bias is identical between data and surrogate fits and obtained a time constant estimate for each neuron together with an uncertainty around this value (Fig 4D) that captured the statistical variability of the estimator (e.g., represented by the log-variance of the estimator).

To systematically aggregate the unequally informative neurons, we used a quasi-Bayesian method that accounts for the bias and uncertainty in the time constant estimation for each neuron (Fig 4B, Methods). Neurons with high uncertainty (low SNR estimate) and relatively weak evidence about the network’s time constant, automatically counted for less contribution. Hence, we did not apply arbitrary criteria to discard neurons with weak autocorrelations, low firing rates or very long timescales, and were able to utilize data from all neurons. We then integrated the evidence from multiple neurons assuming that each estimated time constant per neuron is a noisy version of a single shared network time constant per hemisphere (Fig 4E, Methods). The extracted network time constants were 82 ms (66 ms, 99 ms) for the left ACx and 126 ms (113 ms, 138 ms) for the right ACx (mean, (95% credible interval)). We found that the time constant of the right ACx network was significantly longer (Bayes factor = 0.002, 1 posterior for all neurons compared to a different posterior for each hemisphere). Our results suggest that differences in the recurrent connectivity in superficial layers of the auditory cortices translate into differences in time constants that might support lateralized computation of auditory stimuli.

Our quasi-Bayesian posterior over time constants were consistent with a simple robust average that discarded time constants longer than 300 ms or shorter than 10 ms: 106.1 ms (*n* = 13, SEM = 4.03) for the left and 118.8 ms (*n* = 14, SEM = 3.71) for the right ACx. It is also consistent with the median values, 192.9 ms (*n* = 24) for the left and 250.3 ms (*n* = 27) for the right ACx. Note that the medians are biased, since the outliers are not symmetrically distributed, being bounded by 0 ms from below.

The baseline firing rate of neurons from the right hemisphere tended to be higher. However, weighted linear regression between firing rate and time constant or log-time constant resulted in low r^2 values (0.0167 and 0.0001), and the hemispheric time constant difference explained by the firing rate difference is 7.03 ms, less than ⅕ of the difference. Hence, we rule out that the network time constant difference is due to firing rate differences in the data.

## Discussion

Rodent studies of the last several decades have demonstrated that there are evolutionarily conserved mechanisms of vocalization processing. Behavioral and electrophysiological evidence supports the role of the left ACx in the perception of conspecific vocalizations [6,20,21] and detecting local features in auditory signals, whereas the right ACx specializes in global features [6,7]. Moreover, these processing asymmetries between the auditory cortices were associated with lateralized circuit motifs [6]. In this study, we show a novel neural mechanism whereby hemispheric differences in recurrent connectivity in superficial layers of the ACx translate into distinct temporal integration time windows. We showed that the synaptic organization and network dynamics consistently exhibit lateralization. Using LSPS, we compared the organization and strength of excitatory pathways in the left and right ACx across all cortical layers. For each auditory cortex, we combined the data across cells by building input–output maps. We found significantly stronger intralaminar and interlaminar connectivity between cells in the superficial layers in the right ACx than in the left, suggesting stronger recurrent loops (Fig 2). Moreover, we showed that this enhanced recurrent connectivity in the right ACx leads to stronger recurrent network activity in vitro (Fig 3). Stronger recurrent activity in the right ACx suggests a capacity for enhanced echoic memory: holding a brief memory of auditory signals. This feature would be advantageous to extract auditory information at slower temporal timescales, such as prosody and intonation.

Recurrent connectivity and their positive feedback have long been conjectured to play a key role in persistent neural information processing by lengthening the temporal extent of information representation [12,22,23]. Although there has been a bevy of theories and computational models [24–27], only indirect evidence exists correlating recurrent anatomical structure with observed persistent activity or longer time constants. Previous work has shown that timescales extracted from single neurons’ activity were related to cortical network organization in the primate visual processing hierarchy [28], and to the integration of signals in tasks such as working memory [29], reward guided choice [30,31], and free choice [32]. But there has not been a comparison of the naturally existing recurrent excitatory synaptic circuits and the corresponding network time constants within the same biological model system. In this work, we were able to exploit the lateralized recurrent circuit architecture of the ACx to make the desired direct comparison. The theory suggests that more excitatory recurrent feedback within the superficial layers of the right ACx should give rise to longer network time constant reflected in the neural activity. These differences in recurrent connectivity should impact the temporal structure of both evoked and spontaneous activities [33,34]; however, it is easier to interpret the neural dynamics in the absence of stimulus drive because stimulus dynamics influence the measured time constants. To test the hypothesis, we performed cell-attached recordings in superficial layers of the left and right ACx in awake mice and studied the autocorrelogram of the spontaneous neural activity. Consistent with the connectivity results, our statistical inference showed that the superficial layer network of the right ACx exhibits an approximately 50% longer network time constant relative to the left. To the best of our knowledge, this is the first explicit evidence that excitatory synaptic feedback predicts the spontaneous network dynamics that utilize the natural consistent variations provided by lateralization.

The consensus from decades of human language studies has been that speech decoding is carried out bilaterally with each hemisphere optimized for specific functions. Activity in the left STG has been consistently associated with processing of formant transitions, and perception of speech content is affected by degradation of temporal information; whereas activity in the right STG is associated with processing of intonation contours and is affected by degradation of spectral information [1,35]. However, the underlying neural mechanisms have remained controversial and difficult to prove in humans. One proposed mechanism that could subserve lateralized processing is neuronal ensembles in each hemisphere with different integration time constants: Neurons in the left ACx could preferentially integrate information on shorter timescales, and neurons in the right ACx could preferentially integrate information on longer timescales [4]. Our results support this simultaneous multiscale temporal analysis. Mouse vocalizations are composed of syllables (i.e., a sound unit separated from other sound units by silence [36]), which themselves contain 1 or several pitch trajectories. The duration of individual pitch trajectories within a syllable emitted by adult male mice can range from 30 to 90 ms [37,38]. This range of durations is in line with the distribution of integration time constants we observed in the left ACx (Fig 4E). On the other hand, the right ACx’s time constants were consistent with intersyllabic intervals in mouse vocalizations (140 ms on average; [39]). These hemispheric differences in processing timescales suggest that the left and right ACx are simultaneously processing segmental and suprasegmental information, respectively, and thereby extracting different acoustic cues from the same signal [4]. While the results of our study support asymmetries in temporal integration, our previous work suggests that this is not the only neural mechanism underlying lateralized auditory processing. There are hemispheric differences in the tonotopic organization of auditory cortical circuits and sensitivity to the direction of frequency sweeps, which suggest that there are also lateralized specializations for spectral processing [6]. Overall, the emerging evidence is that there are neural specializations for division labor in both the temporal and spectral domain. In future studies, we plan to use molecular tools to manipulate neural activity in a layer-specific manner to examine how modulating the activity of excitatory networks impacts time constants.

Inhibitory mechanisms have also been proposed to impact network time constants. A recent computational study postulated that the differences in electrophysiological properties between excitatory and inhibitory neurons, as well as differences in the balance between excitation and inhibition, could play a role in hierarchical timescale [40]. Moreover, we have previously reported that inhibitory neuronal populations have lateralized connectivity [41]. In particular, parvalbumin positive (PV+) interneurons in superficial layers of the right ACx can receive excitatory input from deep layers, whereas in the left ACx, they only receive input from superficial layers. This suggests that excitatory input from deep layers in the right ACx could, in principle, modulate the gain of inhibitory PV interneurons in superficial layers. A detailed investigation of lateralized inhibitory mechanisms on network time constants will be conducted in future studies.

As the list of circuit asymmetries grows in the ACx, it will be important to consider how they arise. Asymmetries in genetic programs that guide circuit assembly during development as well as hearing experience are very likely sources of lateralized specializations. These forces can influence the asymmetric formation of both excitatory and inhibitory networks [41]. The observation of greater recurrent connectivity in the right ACx potentially suggests a lower rate of pruning of synaptic contacts during the critical period compared to the left ACx. But it is unclear how layer-specificity would be achieved. In future studies, we plan to dissect cellular and molecular mechanisms of enhanced recurrent connectivity in the right ACx. Although the results from this study were from an animal model, they support prevailing findings from the human literature, provide detailed neural mechanisms, and therefore can potentially offer insight for investigations in humans [42].

## Methods

### Slice preparation and electrophysiology

CBA/J male mice aged P28-51 were used in accordance with the National Institute of Health guidelines, as approved by the City College of New York Institutional Animal Care and Use Committee. Animals were anesthetized and decapitated, and the brains were transferred to a chilled cutting solution composed of (in mM): 110 choline chloride, 25 NaHCO_3_, 25 D-glucose, 11.6 sodium ascorbate, 7 MgCl_2_, 3.1 sodium pyruvate, 2.5 KCl, 1.25 NaH_2_PO_4_, and 0.5 CaCl_2_. We made horizontal slices to map synaptic connectivity along the anterior-posterior axis of the brain where tonotopy is represented in the ACx [43]. We sliced using a 15-degree angle between the blade and the medial-lateral axis so that apical dendrites were parallel to the slice in the ACx. Slices were 300-μm thick and were transferred to artificial cerebrospinal fluid (ACSF) containing (in mM): 127 NaCl, 25 NaHCO_3_, 25 D-glucose, 2.5 KCl, 1 MgCl_2_, 2 CaCl_2_, and 1.25 NaH_2_PO_4_, aerated with 95% O_2_ 5% CO_2_. The slices were incubated at 34°C for 20 to 30 minutes and then kept at room temperature during the experiments. Excitatory neurons located >50 μm below the surface of the slice were visualized using infrared gradient contrast optics and patched with electrodes (6 to 7 MOhm) containing the following intracellular solution (in mM) 128 K-methylsulfate, 4 MgCl, 10 HEPES, 1 EGTA, 4 NaATP, 0.4 NaGTP, 10 Na-phosphocreatine. The pH of the intracellular solution was adjusted to 7.25 and the osmolarity was 300 mOsm. Whole-cell recordings were made using a Multiclamp 700A amplifier (Axon Instruments, Molecular Devices, Sunnyvale, California, United States of America). Excitatory synaptic currents were measured at a holding potential of –70 mV. We used the custom software package ephus ([44] http://www.ephus.org) for instrument control and acquisition written in MATLAB (MathWorks, Natick, Massachusetts, USA).

#### Analysis of IV/FI data

Patched neurons were injected with 11 current pulses (0.5 pA steps) to compute current–voltage relationships (I/V) and frequency–current (F-I) curves. Change in membrane potential for each current step was calculated and added to the resting membrane potential of the cell to compute the I/V relationship. These values were averages across all cells in a population. Responses for the I/V relationships were only up to the first current step that induced spiking in the cell. Firing rate for each current step was also averaged across all cells in a population to calculate F-I curves.

### LSPS by glutamate uncaging

The ACSF for uncaging was supplemented with (in mM): 0.2 nitroindolinyl (NI)-caged glutamate (Tocris), 0.005 CPP (Tocris), and a final concentration of 4 CaCl_2_ and 4 MgCl_2_. For focal photolysis using UV flash to activate the caged glutamate compound, we used a 1 ms light stimulus consisting of 100 pulses from a pulsed UV laser (wavelength, 355 nm with a repetition rate of 100 kHz; DPSS Lasers, Santa Clara, California, USA). These uncaging conditions have been shown to prevent polysynaptic activity [8]; therefore, the synaptic connectivity data shown is monosynaptic. The grid to stimulate the ACx had 16 × 16 uncaging spots with 75 μm spacing, which resulted in a mapping region of 1.125 × 1.125 mm. To avoid revisiting the vicinity of sites recently stimulated, we used a shifting-X pattern that was rotated and/or transposed between map iterations. Each stimulus trial contained a test pulse to measure electrophysiological parameters, and UV flashes were presented every 1 s. The stimulus grid was consistently aligned for each cell recorded as described previously [9]. Briefly, the x axis of the grid was centered on the soma and the y axis was aligned with the second row of the grid placed on the L1/2 border. In previous studies, we confirmed that our slice recordings were performed in A1 (primary ACx) using retrograde tracer DiI and glutamate uncaging in the MGBv (see supplementary figures in [9]).

About 10% of all patched neurons across all layers were presumptive inhibitory cells as evidenced by membrane capacitance <100 pF and high levels of spontaneous synaptic input. These were excluded from the data set. Cells with resting Vm > −50 mV, those that exhibited spontaneous repetitive firing or whose passive membrane properties fluctuated significantly over the course of data collection were also excluded.

#### Disinhibition experiments

For experiments comparing network events in vitro, we used a 2:1 ratio of CaCl_2_ and MgCl_2_, did not block NMDA receptors with CPP, and used 1 to 5 μm of SR95531 (Sigma) to block GABA_A_ receptors. Slices from the left and right ACx were collected from the same animals in these experiments to directly compare the impact of disinhibition. We used an 8 × 16 uncaging grid (75 μm spacing) centered on the cell body. Recordings were performed in the cell-attached configuration. All other photostimulation parameters were the same as described above.

### Analysis of LSPS data

Analysis was conducted as described previously [9]. Briefly, the mean current amplitude of synaptic events was calculated in the 50-ms epoch after the direct response time window (7.5 ms after UV stimulus). Direct responses triggered by stimulation around somata were excluded in our analyses. We recorded 2 to 4 maps for each cell to create an average input map, and these average maps were used for group averages and for all analyses. Like the individual maps, population maps were aligned with respect to the soma on the x axis and the L1/L2 boundary on the y axis. Pixels in the population maps where direct responses were recorded in >50% of cells, i.e., areas proximal to the cell body, were excluded from the analysis.

To create input–output functions from the map data, average maps for each cell were summed over either the antero-posterior axis or the laminar axis to yield 1 × 16 vectors of input strength versus location. These vectors were then binned according to laminar or antero-posterior location, respectively, and combined to yield n × 16 or 16 × n matrices relating presynaptic input to postsynaptic soma location (“output”), where *n* = number of bins as reported in the individual figure legends. The matrices were 2D-interpolated for display purposes.

To analyze network events in disinhibition experiments, we calculated the number of action potentials evoked per stimulation site and the duration of network events. All network events began with the firing of an action potential, and the event duration was calculated as the return of the membrane potential back to the mean of the pre-stimulus baseline period (±1.5 std).

*Statistical significance between hemispheres* for population map values was computed pixel-by-pixel, with the average map value for each cell being considered a single data point. Comparisons were made via 2-tailed, unpaired *t* tests. For comparison, randomized data sets were generated by pooling all maps from both hemispheres for each layer and drawing maps at random to give 2 pools with the same *n* as the original data sets.

### In vivo recordings

#### Subjects and surgery

A total of 14 CBA/J mice, aged P30-60, were used in accordance with the National Institute of Health guidelines, as approved by the City College of New York Institutional Animal Care and Use Committee. We administered ketamine (75 mg kg^−1^)/medetomidine (0.5 mg kg^−1^) before a stereotactic surgery was performed to allow for awake recordings. The scalp from the entire top of the skull was removed to reveal bregma and lambda, barely exposing the muscle of the temporal bone. A metal plate was bound to the exposed bone via 2 layers of Metabond and a single layer of Vitribond, followed by a covering of dental cement. Following administration of ketamine (75 mg kg^−1^)/medetomidine (0.5 mg kg^−1^), mice were head-fixed on a freely rotating wheel inside a soundproof chamber. We made a craniotomy and durotomy over the auditory cortex, centered at 2.5 mm posterior and 4 mm lateral to bregma. Craniotomies were 0.1 to 0.2 mm in diameter to provide stability to our recordings. Mice were allowed at least 24 hours post-surgery to recover before any recording sessions began.

#### Electrophysiology

We recorded from the left and right ACx of awake mice. During the recording sessions, we targeted neurons in superficial layers (L2/3, L4; 150 to 500 μm below cortical surface) using the standard blind cell-attached technique [45]. One hemisphere was recorded per animal. Electrodes were pulled from a glass borosilicate filament and filled with either physiological saline (0.9% NaCl) or intracellular solution ((in mM) 128 K-methylsulfate, 4 MgCl, 10 HEPES, 1 EGTA, 4 NaATP, 0.3 NaGTP, 10 Na-phos-phocreatine) and had resistances between 4 to 8 MOhms. Recordings were obtained using Axopatch 200B (Axon Instruments) and custom electrophysiological data acquisition software (exper, by Tomas Hromadka) written in MATLAB (Mathworks). We detected the presence of cells in the cortex based on changes in pipette resistance.

A soundproof chamber was used to conduct all recordings. We used a custom-built real-time Linux system (200 kHz sampling rate) driving a high-end Lynx L22 audio card (Lynx Studio Technology, Newport Beach, California, USA) with an ED1 electrostatic speaker (Tucker-Davis Technologies, Alachua, Florida, USA) in free-field configuration (speaker located 6 inches lateral to, and facing, the contralateral ear). The stimuli were generated with custom MATLAB scripts. To compute tuning curves of our cells’ best frequency, we used a set of pure tones that lasted 100 ms long of 16 different frequencies at 3 intensity levels (20dB, 50dB, and 80dB). Tuning curves and timing of tone-evoked responses (40 to 50 ms from stimulus presentation) were used to ensure that the position of our recordings were within the primary ACx.

### Time constant posterior distributions

#### Link between recurrent anatomy and time constant

The causal connection between recurrent connectivity and time constant is based on the intuitive fact that excitatory feedback increases time constant in the first-order approximation of a system. In particular, consider a first-order linear dynamical system that represents the neural population with no feedback with time constant $\tau :\;\frac{dx}{dt}=-\frac{1}{\tau }\cdot x+\eta$, where *x*(*t*) represents the 1D subspace of the population activity and *η* denotes white noise representing noise and input drive. The autocorrelation of this system has the form: $\mathrm{acf}(lag)=a\;e^{-\frac{\left|lag\right|}{\tau }}$. Since we fit the autocorrelation function with a single τ, it would correspond to the dominant time constant expressed in the neuron’s activity. We assume that the neural autocorrelation directly reflects the time constant of the population (i.e., the network time constant). Now if there is positive feedback in the first-order linear system, we get: $\frac{dx}{dt}=-\frac{1}{\tau }\cdot x+g\cdot x+\eta$, where 0<*g*<1/τ is a positive constant that captures the strength of the feedback of the population. In this simplified form, we can see that the effective time constant increases as a function of *g*, since $\frac{dx}{dt}=-\left(\frac{1}{\tau }-g\right)\cdot x+\eta =-\frac{1}{\tau_{\mathrm{eff}}}\cdot x+\eta$, where the effective time constant with the recurrence is $\tau_{eff}=\frac{\tau }{1\;-\tau \cdot g}>\tau$. Hence, under these first-order approximations, excitatory feedback is expected to increase the time constant of spontaneous activity.

#### Autocorrelation and single neuron time constant estimation

For this analysis, we used inter-trial interval recordings in which no auditory stimulus was played, and we were blind to the hemispheric identity of the data. Each inter-trial interval started at least 100 ms after the end of the stimulus and of duration 1,540 ms. We binned the recordings in *Δ* = 20 ms bins and computed the unbiased autocorrelation function:

$$\hat{\mathrm{acf}}(l)=\frac{1}{N_{\mathrm{trial}}}\sum_{n=1}^{N_{\mathrm{trial}}}\frac{1}{T_{n}-l+1}\sum_{t=1}^{T_{n}}x_{n}(t)x_{n}(t-l),$$

where *l* denotes the *l*-th binned lag, *x*_*n*_(*t*) is the binned spontaneous spike trains for trial *n*, *T*_*n*_ is the number of time bins for the corresponding trial, and *N*_trial_ is the total number of trials.

For each neuron, we performed nonlinear least squares fits to the estimated autocorrelation functions, exponential decays of the form

$$C(l)=a\cdot e^{-\frac{l}{\hat{\tau }}}+b$$ (1)

with multiple initializations to extract the values of the parameters $\hat{\tau }$ and *a*. We used the autocorrelation function starting from 20 ms up to 760 ms binned at 20 ms. Disregarding the first 20 ms of the autocorrelation removes the dominant very short time constant component in the data. We assumed the process is stationary and fixed the value of *b* to (*λΔ*)^2^ where *λ* is the mean firing rate. We then estimated the bias and variance of the estimated time constant $\hat{\tau }$ for each neuron using the DG model.

#### Dichotomized Gaussian model

The DG model is a spiking generative model that can capture correlation structures in spiking neural data [46]. Sample (binarized) spike trains *X*(*t*) were generated by thresholding samples of a latent multivariate Gaussian time series *V*(*t*)

X(t)=1iffV(t)>0withV∼N(γ,Σ)

with *Σ*(*t*, *t*) = 1 without loss of generality. Assuming the process is stationary, *γ* is a constant and the time covariance matrix *Σ*(*t*, *s*) = *Σ*(*t*−*s*) is only a function of the lag between time points. The latent parameters *γ* and *Σ* were obtained from the spiking rate *λ* per bin and autocorrelation *C* using

$$\gamma =\Phi^{-1}(\lambda )$$

$$C(t-s)-\Phi_{2}(\lambda ,\lambda ,\Sigma (t-s))=0,$$

where *Φ* and *Φ*_2_ denote the cumulative distribution of the standard normal and the bivariate normal distributions with given correlation [46]. Since we use the exponential decay form for the autocorrelation, *Σ*(*t*−*s*) is monotonically decaying as function of the lag (*t*−*s*), there is a unique solution for *γ* constrained to be in the range (−1, 1). In this way, the DG model can capture specific spiking rates and temporal autocorrelations in the data.

#### Bias and variance estimation

For each neuron, we generated 400 sets of surrogate data replicating the recording conditions and using the DG model with the exponential autocorrelation of Eq (1) and the extracted parameters $\hat{\tau }$ and *a*. We repeated the estimation procedure for each set and obtained a set of surrogate time constants $\left\{\tau_{1}^{DG},\tau_{2}^{DG},\ldots ,\tau_{400}^{DG}\right\}$. We assumed these values are observations from a log-normal distribution ${log(\tau }_{i}^{DG})∼Ν(log(\hat{\tau })+bias,\sigma^{2})$ and we estimated their mean and variance by maximum likelihood. The difference between the estimated mean $E[\log(\tau^{DG})]$ and $\log(\hat{\tau })$ is an estimate of the bias of our estimator in our procedure and *σ*^2^ represents the uncertainty of the estimate.

#### Evidence integration

We assumed that the bias corrected time constants $log({\hat{\tau }}_{i}^{u})=log({\hat{\tau }}_{i})-{bias}_{i}$ obtained from the neurons in each hemisphere come from a log-normal distribution $log({\hat{\tau }}_{i}^{u})∼Ν(log(\tau ),\sigma_{i}^{2})$ with *τ* the network’s time constant and $\sigma_{i}^{2}$ the variance of log time constant estimated for each neuron. Assuming a uniform prior for *τ*, we integrate the evidence from the observations of each hemisphere into the posterior distributions by computing $P(\tau |\{\tau_{i}\})\propto \prod_{i}P(\tau_{i}|\tau ,\sigma_{i}^{2})$.

## Supporting information

S1 Fig No hemispheric differences in measured membrane properties. Whole-cell recordings were performed in superficial layers (L2-4) of the left and right ACx at room temperature (see Methods for experimental details). A series of current steps were injected to test the subthreshold and suprathreshold membrane properties of excitatory neurons. Left panel shows the relationship between current injected and evoked membrane response measured at steady state. Right panel is the relationship between current injected and firing rate, also measured at steady state. The data underlying all the plots in this figure are included in S1 Data.
(TIF)

S1 Data Excel spreadsheets containing the quantitative data for each experiment as described in the results and figure legends. (XLSX)

S1 File The 45 figures in this document contain data from all the cells recorded in vivo and analyzed in Fig 4. The blue raster panels correspond to the data recorded and the pink to the DG model. Data from the left ACx have files named Calyx and the right ACx Thelo.
(PDF)

## Abbreviations

ACSF: artificial cerebrospinal fluid
ACx: auditory cortex
DG: dichotomized Gaussian
LSPS: laser scanning photostimulation
SNR: signal-to-noise-ratio
STG: superior temporal gyrus
