# Supplementary figures and images for: Differences in temporal processing speeds between the right and left auditory cortex reflect the strength of recurrent synaptic connectivity
Source: PLoS Biol. 2022 Oct 21;20(10):e3001803. doi: 10.1371/journal.pbio.3001803 (PMC9629599; doi:10.1371/journal.pbio.3001803)

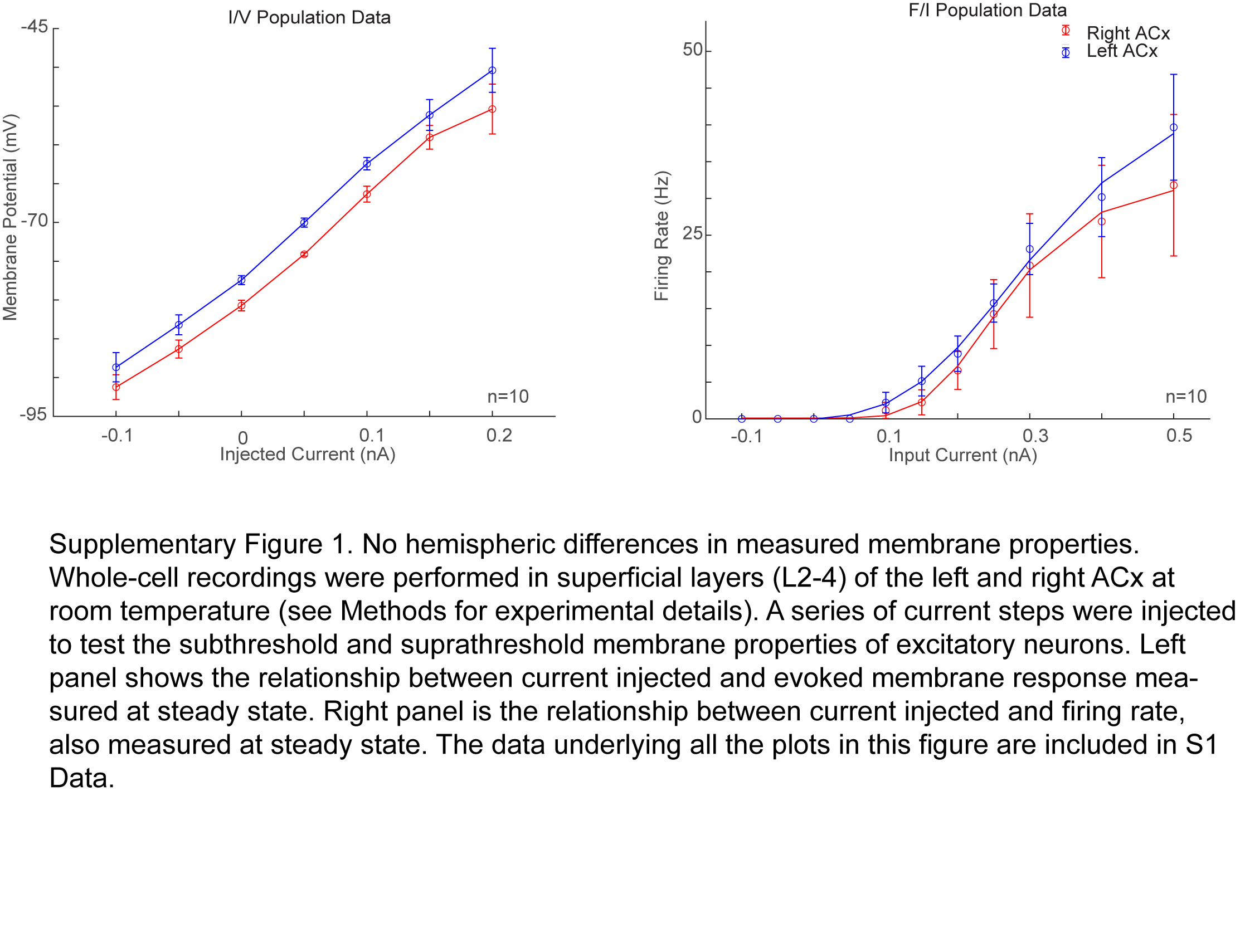

Supplement: S1 Fig — Whole-cell recordings were performed in superficial layers (L2-4) of the left and right ACx at room temperature (see Methods for experimental details). A series of current steps were injected to test the subthreshold and suprathreshold membrane properties of excitatory neurons. Left panel shows the relationship between current injected and evoked membrane response measured at steady state. Right panel is the relationship between current injected and firing rate, also measured at steady state. The data underlying all the plots in this figure are included in S1 Data. (TIF) [file pbio.3001803.s001.tif]
