# Supplementary material for: Differences in temporal processing speeds between the right and left auditory cortex reflect the strength of recurrent synaptic connectivity
Source: PLoS Biol. 2022 Oct 21;20(10):e3001803. doi: 10.1371/journal.pbio.3001803 (PMC9629599; doi:10.1371/journal.pbio.3001803)
Supplement: S1 File — The blue raster panels correspond to the data recorded and the pink to the DG model. Data from the left ACx have files named Calyx and the right ACx Thelo. (PDF) [file pbio.3001803.s003.pdf]

Supplementary figures

The 45 figures in this document contain data from all the cells recorded in vivo and analyzed in figure 4. The blue raster panels correspond to the data recorded, and the pink to the DG model. Data from the left ACx have files named Calyx, and the right ACx Thelo.

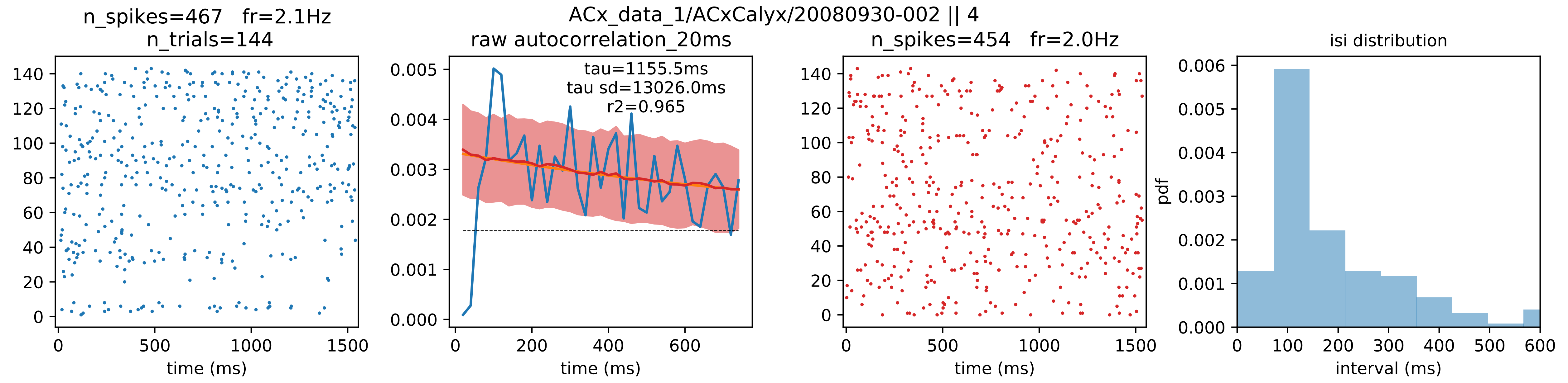

ACx_data_1/ACxCalyx/20080930-002 || 4
n_spikes=467 fr=2.1Hz
n_trials=144
time (ms)
raw autocorrelation_20ms
tau=1155.5ms
tau sd=13026.0ms
r2=0.965
time (ms)
n_spikes=454 fr=2.0Hz
time (ms)
isi distribution
pdf
interval (ms)

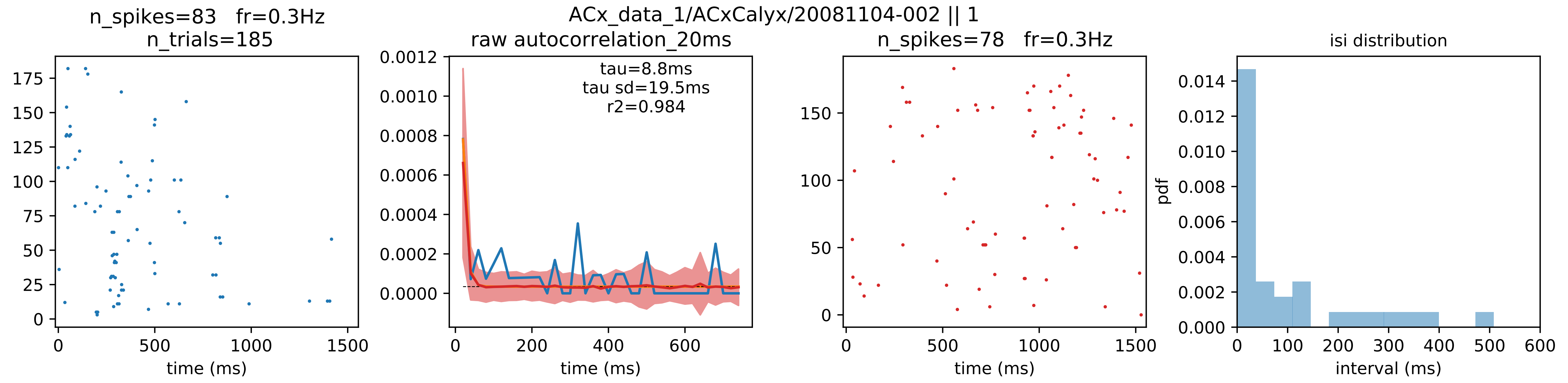
ACx_data_1/ACxCalyx/20081104-002 || 1
n_spikes=83 fr=0.3Hz
n_trials=185
time (ms)
raw autocorrelation_20ms
tau=8.8ms
tau sd=19.5ms
r2=0.984
time (ms)
n_spikes=78 fr=0.3Hz
time (ms)
isi distribution
pdf
interval (ms)

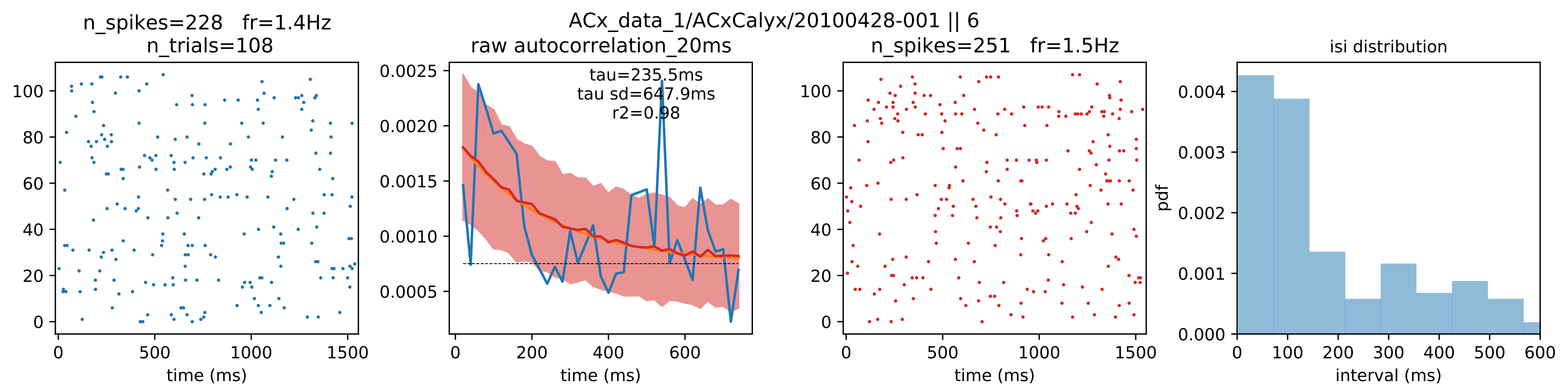

ACx_data_1/ACxCalyx/20100428-001 || 6
n_spikes=228 fr=1.4Hz
n_trials=108
time (ms)
raw autocorrelation_20ms
tau=235.5ms
tau sd=647.9ms
r2=0.98
time (ms)
n_spikes=251 fr=1.5Hz
time (ms)
isi distribution
pdf
interval (ms)

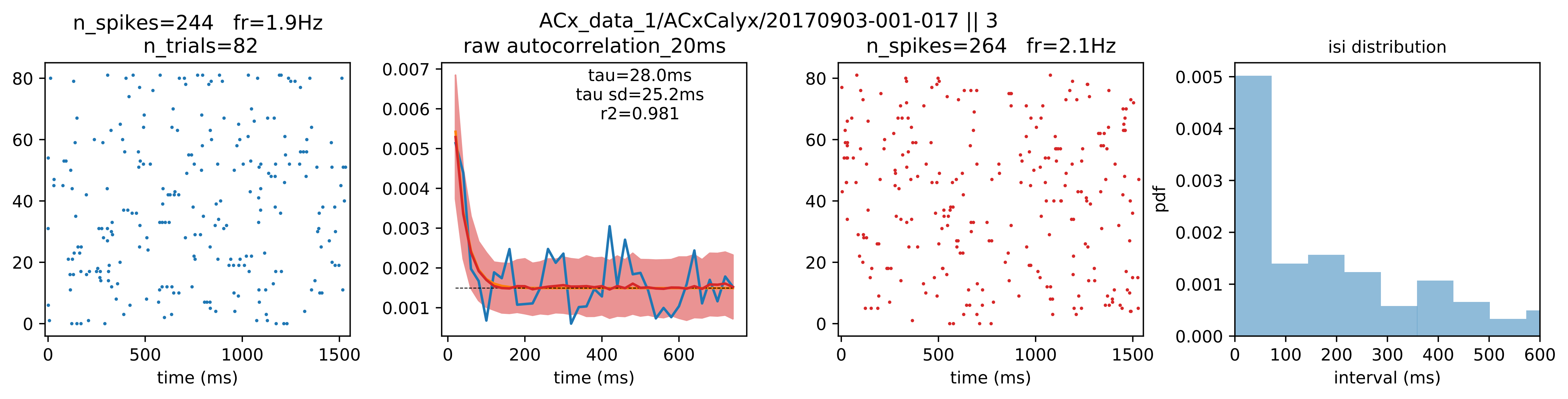
ACx_data_1/ACxCalyx/20170903-001-017 || 3
n_spikes=244 fr=1.9Hz
n_trials=82
time (ms)
raw autocorrelation_20ms
tau=28.0ms
tau sd=25.2ms
r2=0.981
time (ms)
n_spikes=264 fr=2.1Hz
time (ms)
isi distribution
pdf
interval (ms)

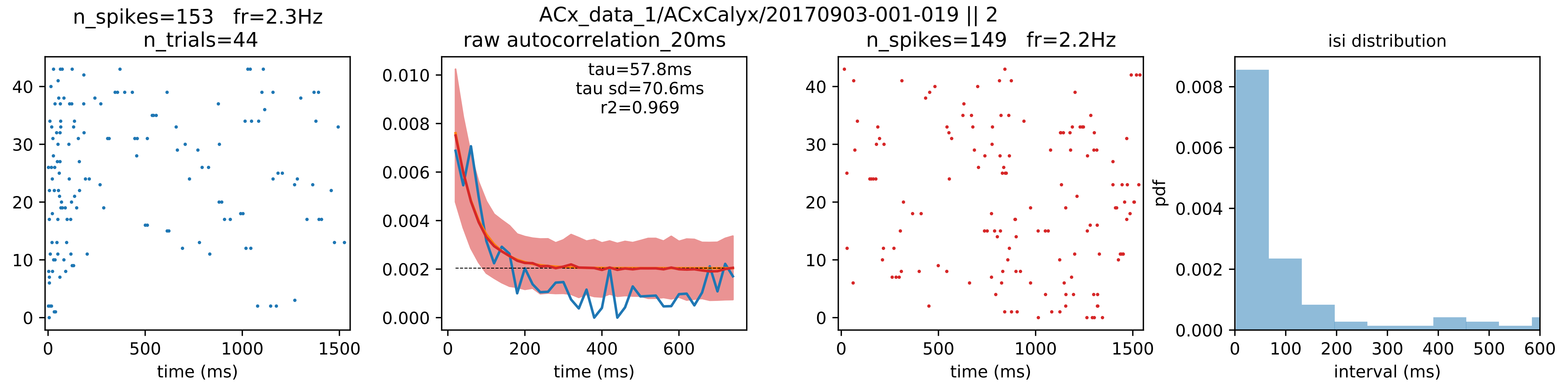

ACx_data_1/ACxCalyx/20170903-001-019 || 2
n_spikes=153 fr=2.3Hz
n_trials=44
time (ms)
raw autocorrelation_20ms
tau=57.8ms
tau sd=70.6ms
r2=0.969
time (ms)
n_spikes=149 fr=2.2Hz
time (ms)
isi distribution
pdf
interval (ms)

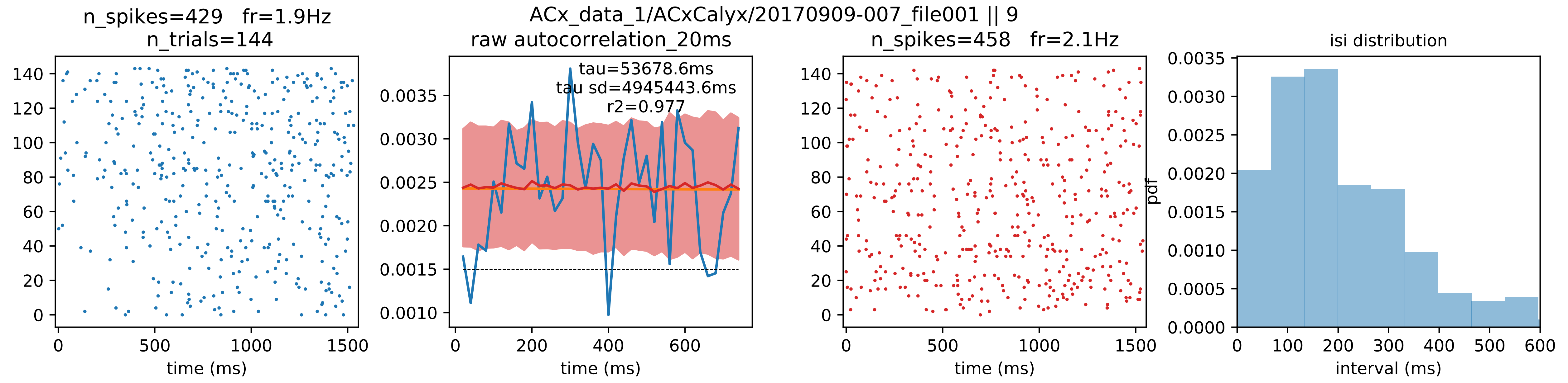

ACx_data_1/ACxCalyx/20170909-007_file001 || 9
n_spikes=429 fr=1.9Hz
n_trials=144
time (ms)
raw autocorrelation_20ms
tau=53678.6ms
tau sd=4945443.6ms
r2=0.977
time (ms)
n_spikes=458 fr=2.1Hz
time (ms)
isi distribution
pdf
interval (ms)

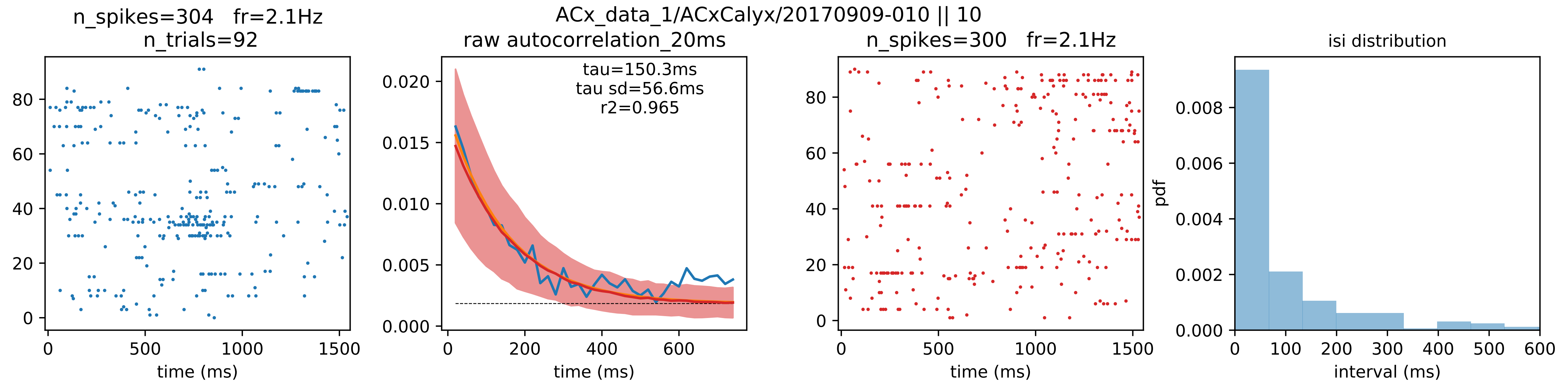
ACx_data_1/ACxCalyx/20170909-010 || 10
n_spikes=304 fr=2.1Hz
n_trials=92
time (ms)
raw autocorrelation_20ms
tau=150.3ms
tau sd=56.6ms
r2=0.965
time (ms)
n_spikes=300 fr=2.1Hz
time (ms)
isi distribution
pdf
interval (ms)

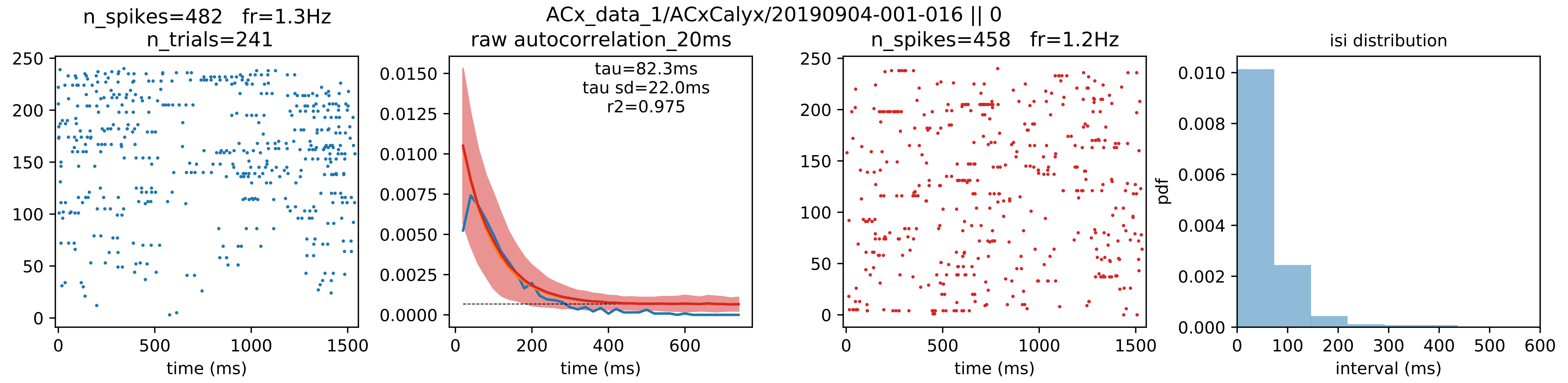

ACx_data_1/ACxCalyx/20190904-001-016 || 0
n_spikes=482 fr=1.3Hz
n_trials=241
time (ms)
raw autocorrelation_20ms
tau=82.3ms
tau sd=22.0ms
r2=0.975
time (ms)
n_spikes=458 fr=1.2Hz
time (ms)
isi distribution
pdf
interval (ms)

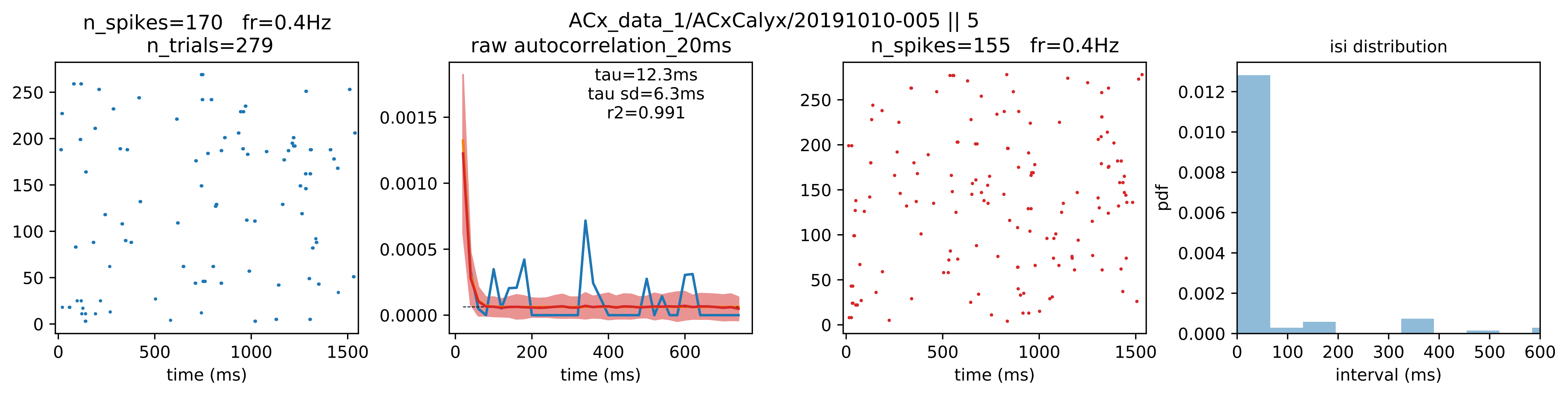
ACx_data_1/ACxCalyx/20191010-005 || 5
n_spikes=170 fr=0.4Hz
n_trials=279
time (ms)
raw autocorrelation_20ms
tau=12.3ms
tau sd=6.3ms
r2=0.991
time (ms)
n_spikes=155 fr=0.4Hz
time (ms)
isi distribution
pdf
interval (ms)

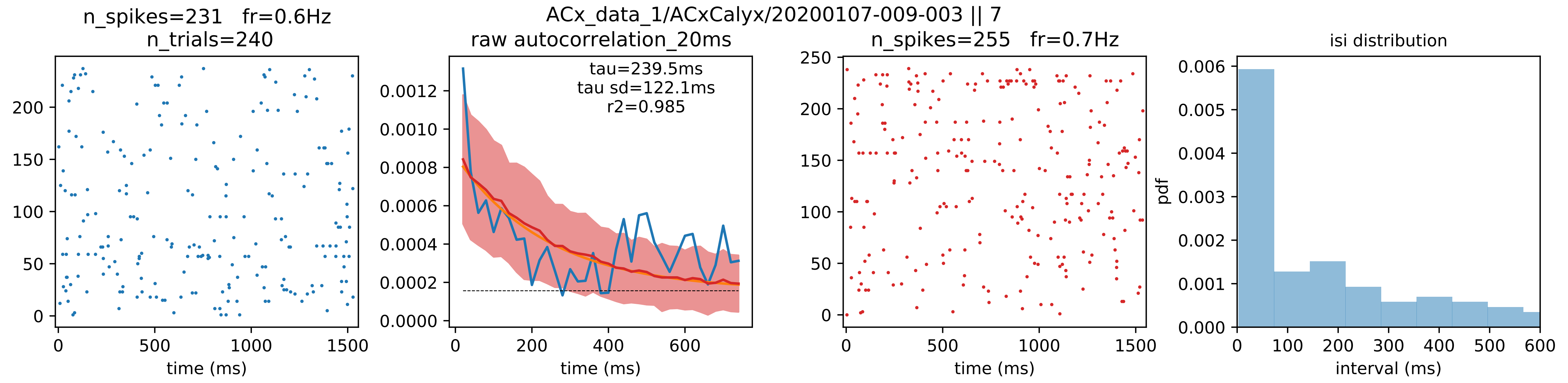

ACx_data_1/ACxCalyx/20200107-009-003 || 7
n_spikes=231 fr=0.6Hz
n_trials=240
time (ms)
raw autocorrelation_20ms
tau=239.5ms
tau sd=122.1ms
r2=0.985
time (ms)
n_spikes=255 fr=0.7Hz
time (ms)
isi distribution
pdf
interval (ms)

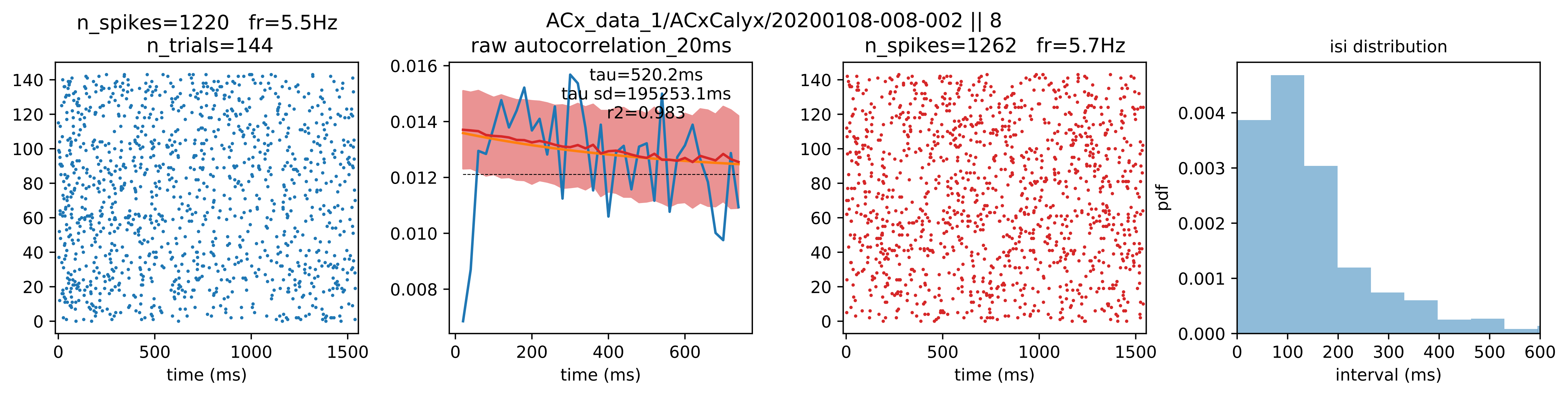

ACx_data_1/ACxCalyx/20200108-008-002 || 8
n_spikes=1220 fr=5.5Hz
n_trials=144
time (ms)
raw autocorrelation_20ms
tau=520.2ms
tau sd=195253.1ms
r2=0.983
time (ms)
n_spikes=1262 fr=5.7Hz
time (ms)
isi distribution
pdf
interval (ms)

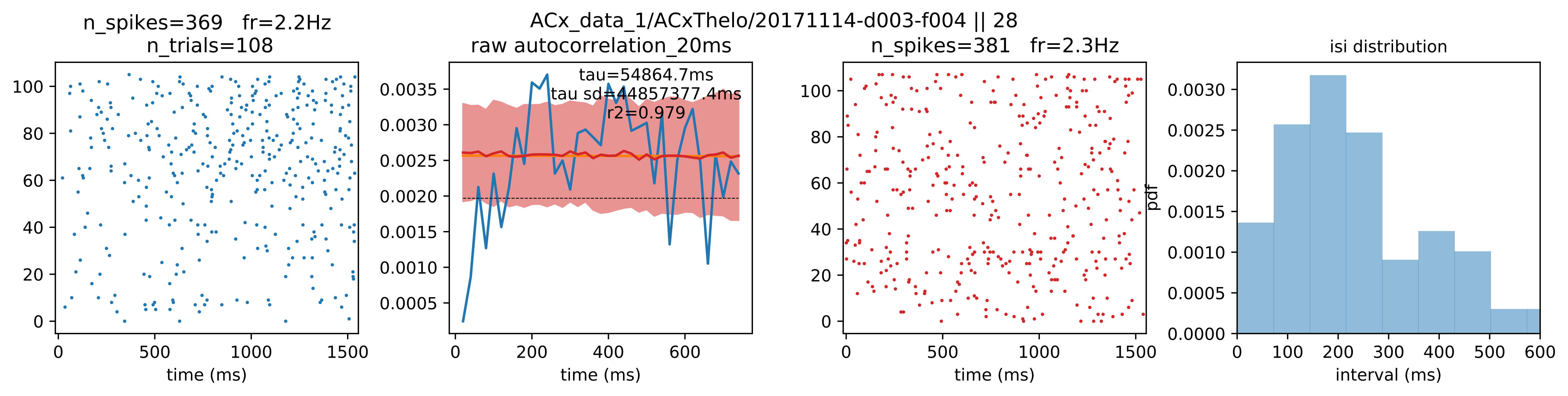

ACx_data_1/ACxThelo/20171114-d003-f004 || 28
n_spikes=369 fr=2.2Hz
n_trials=108
time (ms)
raw autocorrelation_20ms
tau=54864.7ms
tau sd=44857377.4ms
r2=0.979
time (ms)
n_spikes=381 fr=2.3Hz
time (ms)
isi distribution
pdf
interval (ms)

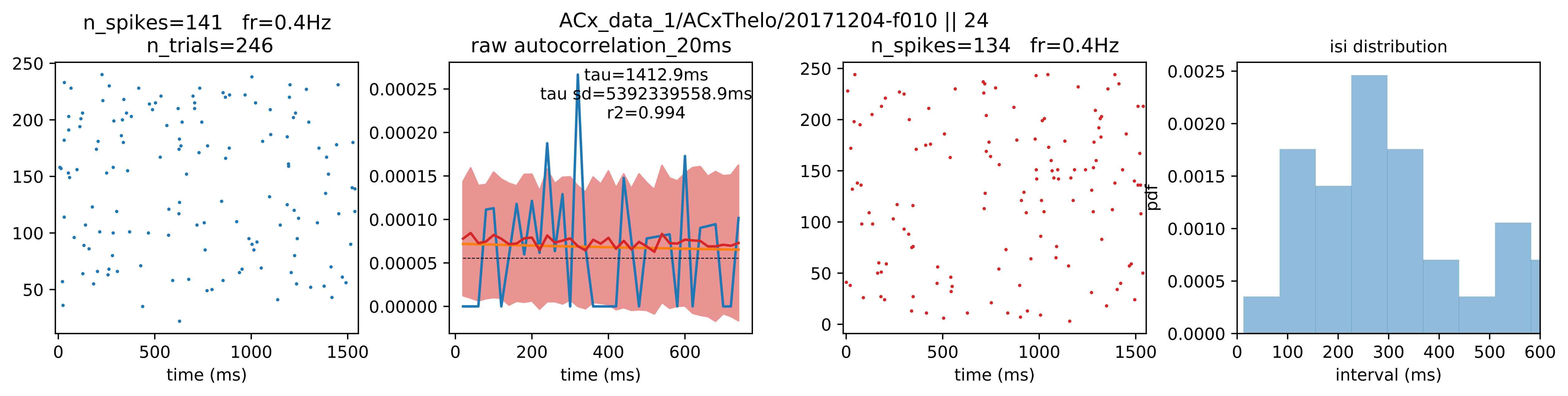
ACx_data_1/ACxThelo/20171204-f010 || 24
n_spikes=141 fr=0.4Hz
n_trials=246
raw autocorrelation_20ms
tau=1412.9ms
tau sd=5392339558.9ms
r2=0.994
n_spikes=134 fr=0.4Hz
isi distribution
time (ms)
time (ms)
time (ms)
interval (ms)
pdf

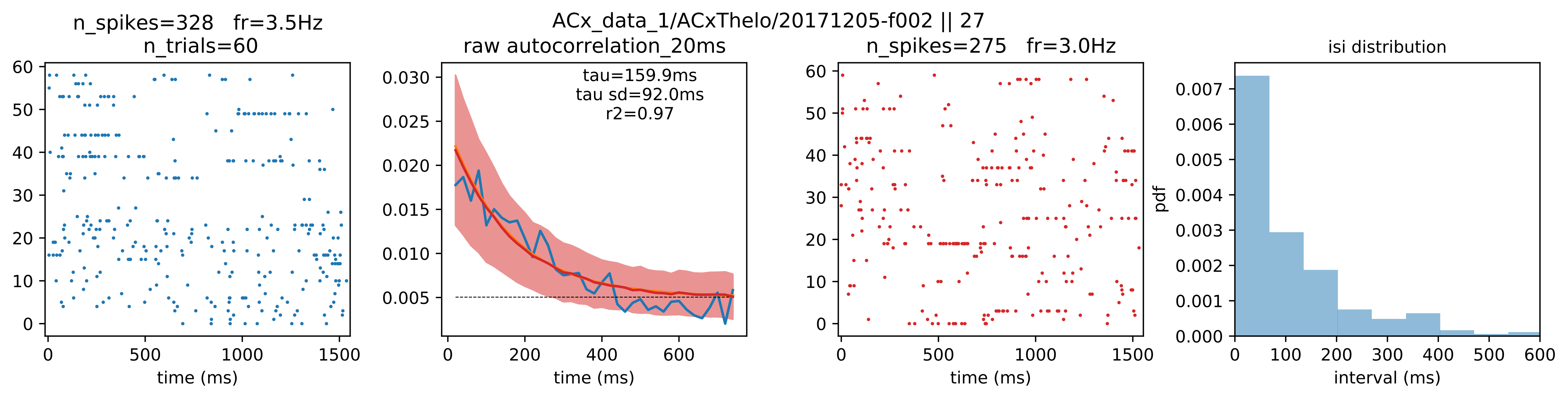
ACx_data_1/ACxThelo/20171205-f002 || 27
n_spikes=328 fr=3.5Hz
n_trials=60
time (ms)
raw autocorrelation_20ms
tau=159.9ms
tau sd=92.0ms
r2=0.97
time (ms)
n_spikes=275 fr=3.0Hz
time (ms)
isi distribution
pdf
interval (ms)

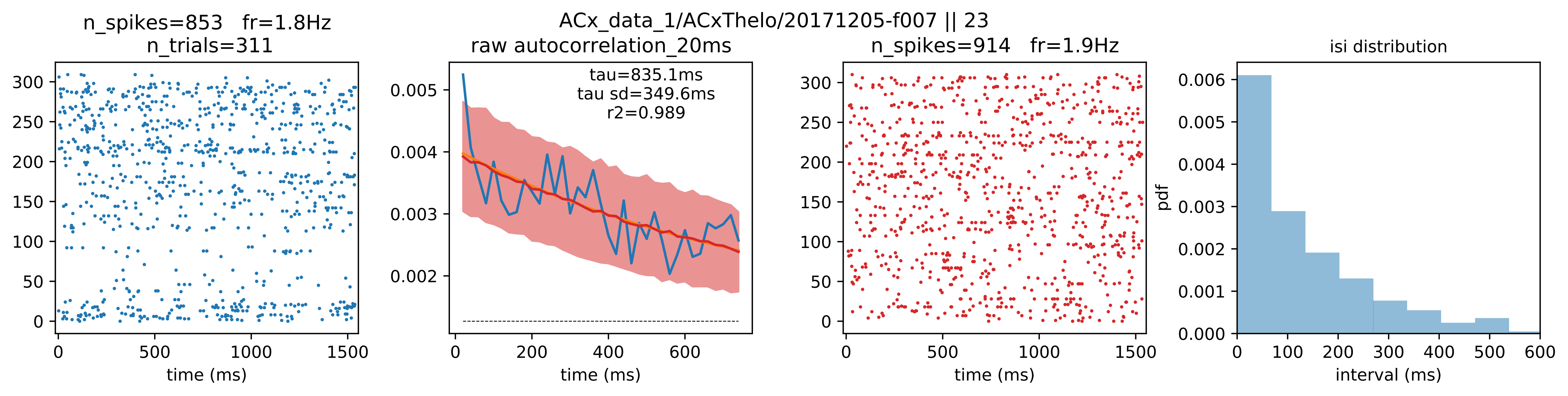
ACx_data_1/ACxThelo/20171205-f007 || 23
n_spikes=853 fr=1.8Hz
n_trials=311
time (ms)
raw autocorrelation_20ms
tau=835.1ms
tau sd=349.6ms
r2=0.989
time (ms)
n_spikes=914 fr=1.9Hz
time (ms)
isi distribution
pdf
interval (ms)

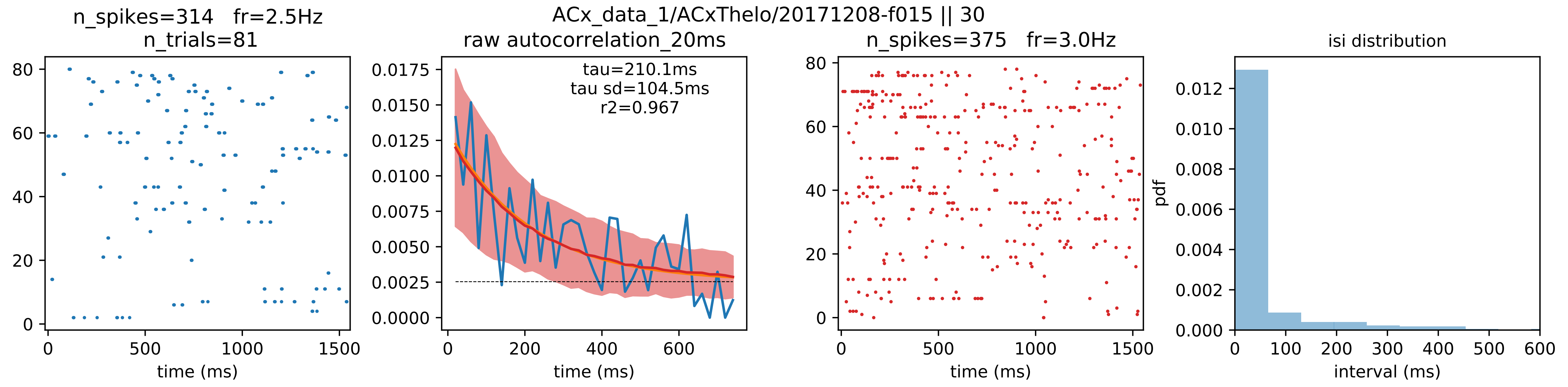

ACx_data_1/ACxThelo/20171208-f015 || 30
n_spikes=314 fr=2.5Hz
n_trials=81
time (ms)
raw autocorrelation_20ms
tau=210.1ms
tau sd=104.5ms
r2=0.967
time (ms)
n_spikes=375 fr=3.0Hz
time (ms)
isi distribution
pdf
interval (ms)

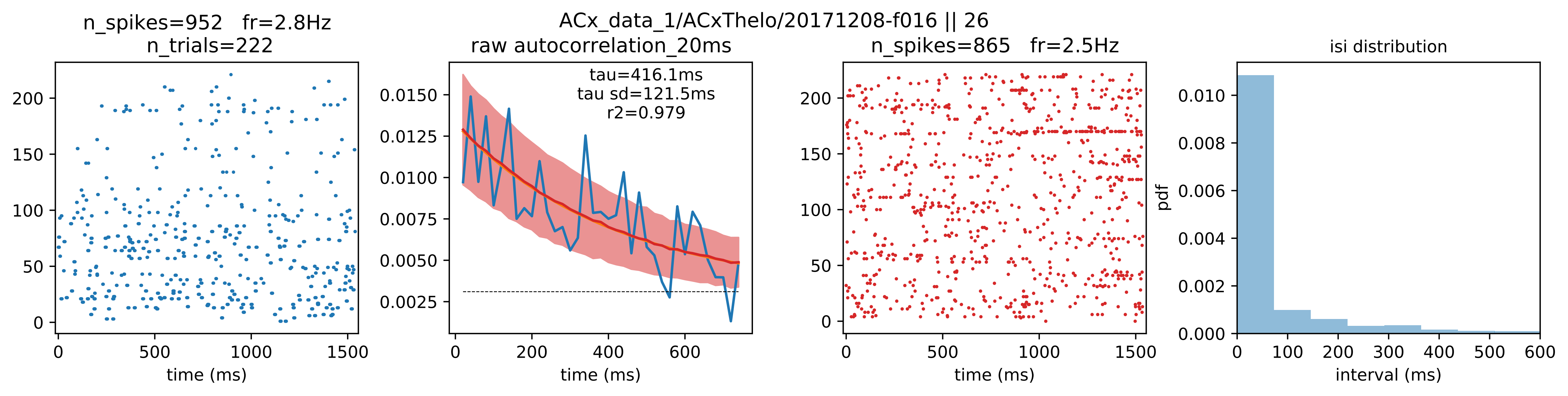
ACx_data_1/ACxThelo/20171208-f016 || 26
n_spikes=952 fr=2.8Hz
n_trials=222
time (ms)
raw autocorrelation_20ms
tau=416.1ms
tau sd=121.5ms
r2=0.979
time (ms)
n_spikes=865 fr=2.5Hz
time (ms)
isi distribution
pdf
interval (ms)

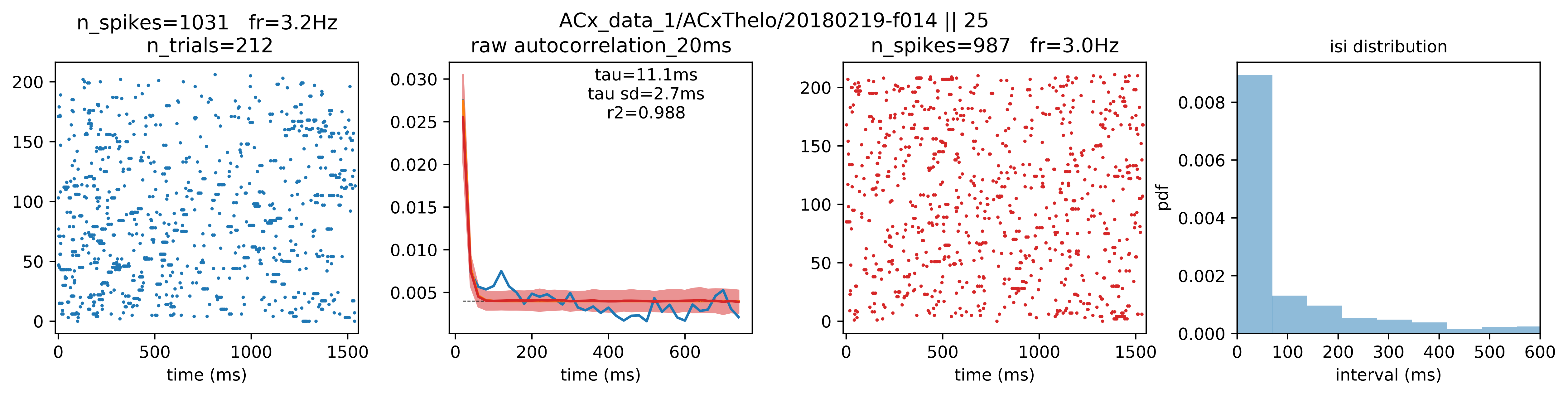
ACx_data_1/ACxThelo/20180219-f014 || 25
n_spikes=1031 fr=3.2Hz
n_trials=212
time (ms)
raw autocorrelation_20ms
tau=11.1ms
tau sd=2.7ms
r2=0.988
time (ms)
n_spikes=987 fr=3.0Hz
time (ms)
isi distribution
pdf
interval (ms)

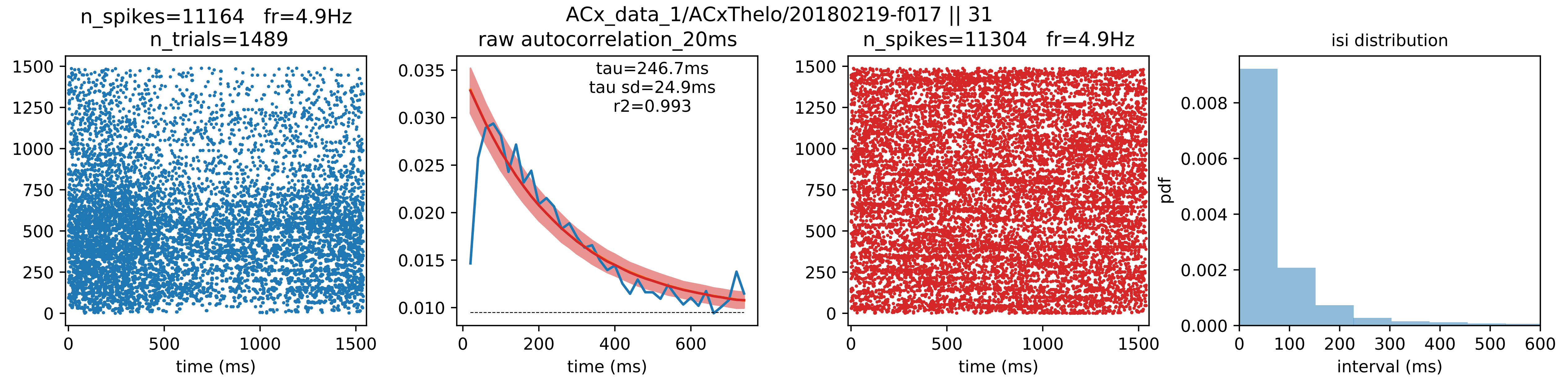

ACx_data_1/ACxThelo/20180219-f017 || 31
n_spikes=11164 fr=4.9Hz
n_trials=1489
time (ms)
raw autocorrelation_20ms
tau=246.7ms
tau sd=24.9ms
r2=0.993
time (ms)
n_spikes=11304 fr=4.9Hz
time (ms)
isi distribution
pdf
interval (ms)

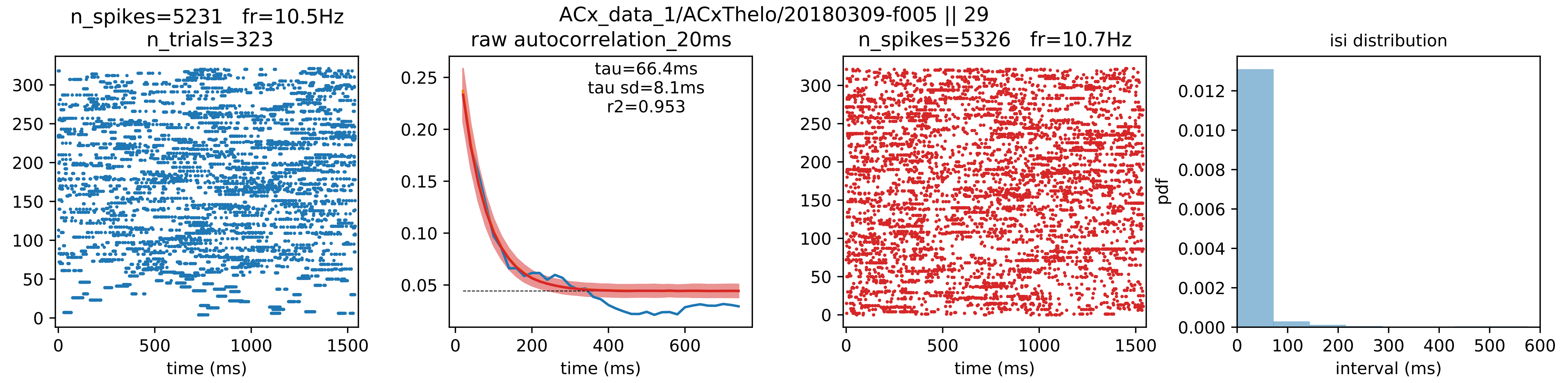

ACx_data_1/ACxThelo/20180309-f005 || 29
n_spikes=5231 fr=10.5Hz
n_trials=323
time (ms)
raw autocorrelation_20ms
tau=66.4ms
tau sd=8.1ms
r2=0.953
time (ms)
n_spikes=5326 fr=10.7Hz
time (ms)
isi distribution
pdf
interval (ms)

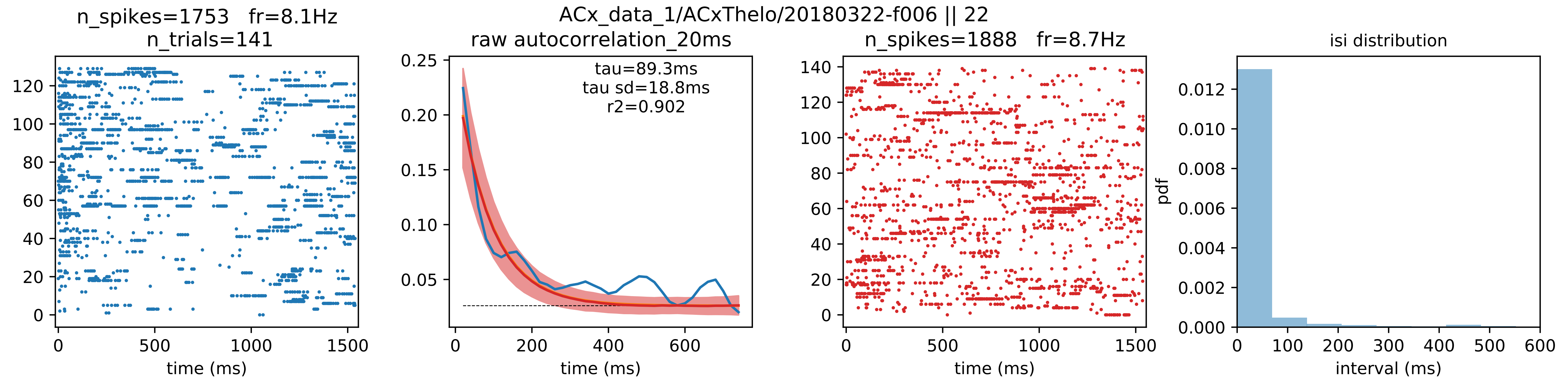

ACx_data_1/ACxThelo/20180322-f006 || 22
n_spikes=1753 fr=8.1Hz
n_trials=141
time (ms)
raw autocorrelation_20ms
tau=89.3ms
tau sd=18.8ms
r2=0.902
time (ms)
n_spikes=1888 fr=8.7Hz
time (ms)
isi distribution
pdf
interval (ms)

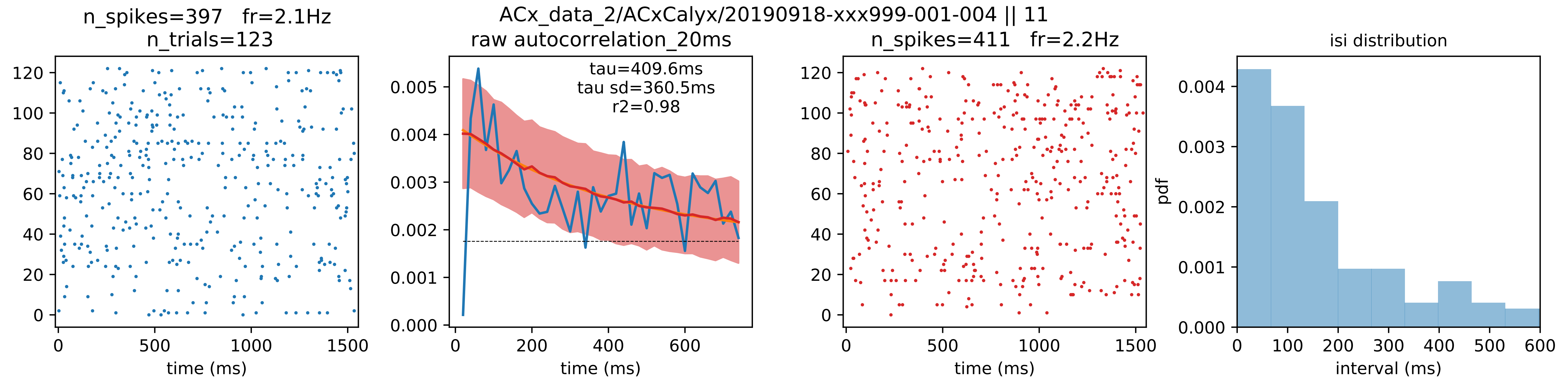

ACx_data_2/ACxCalyx/20190918-xxx999-001-004 || 11
n_spikes=397 fr=2.1Hz
n_trials=123
time (ms)
raw autocorrelation_20ms
tau=409.6ms
tau sd=360.5ms
r2=0.98
time (ms)
n_spikes=411 fr=2.2Hz
time (ms)
isi distribution
pdf
interval (ms)

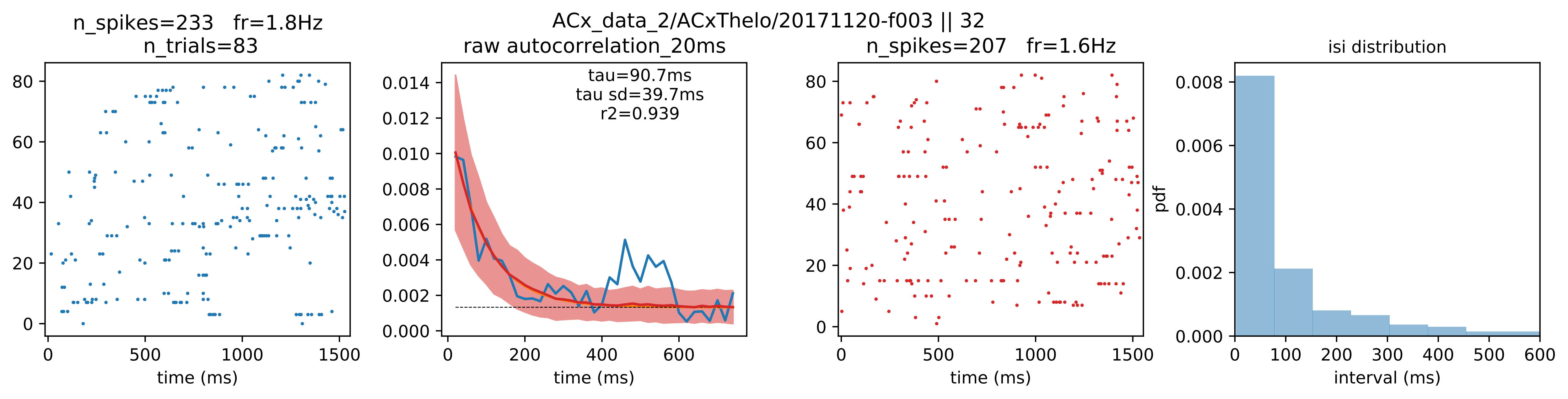

ACx_data_2/ACxThelo/20171120-f003 || 32
n_spikes=233 fr=1.8Hz
n_trials=83
time (ms)
raw autocorrelation_20ms
tau=90.7ms
tau sd=39.7ms
r2=0.939
time (ms)
n_spikes=207 fr=1.6Hz
time (ms)
isi distribution
pdf
interval (ms)

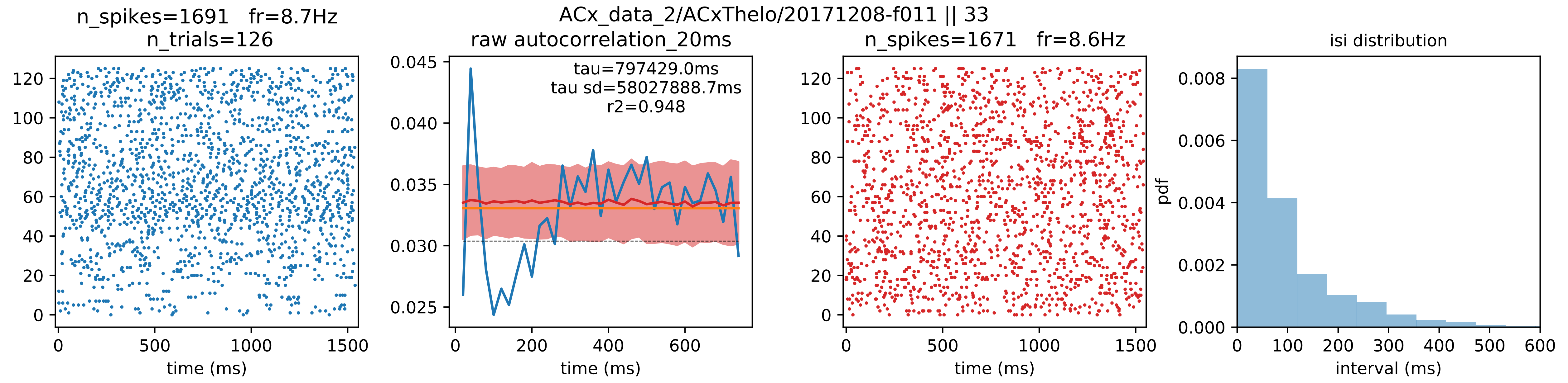

ACx_data_2/ACxThelo/20171208-f011 || 33
n_spikes=1691 fr=8.7Hz
n_trials=126
time (ms)
raw autocorrelation_20ms
tau=797429.0ms
tau sd=58027888.7ms
r2=0.948
time (ms)
n_spikes=1671 fr=8.6Hz
time (ms)
isi distribution
pdf
interval (ms)

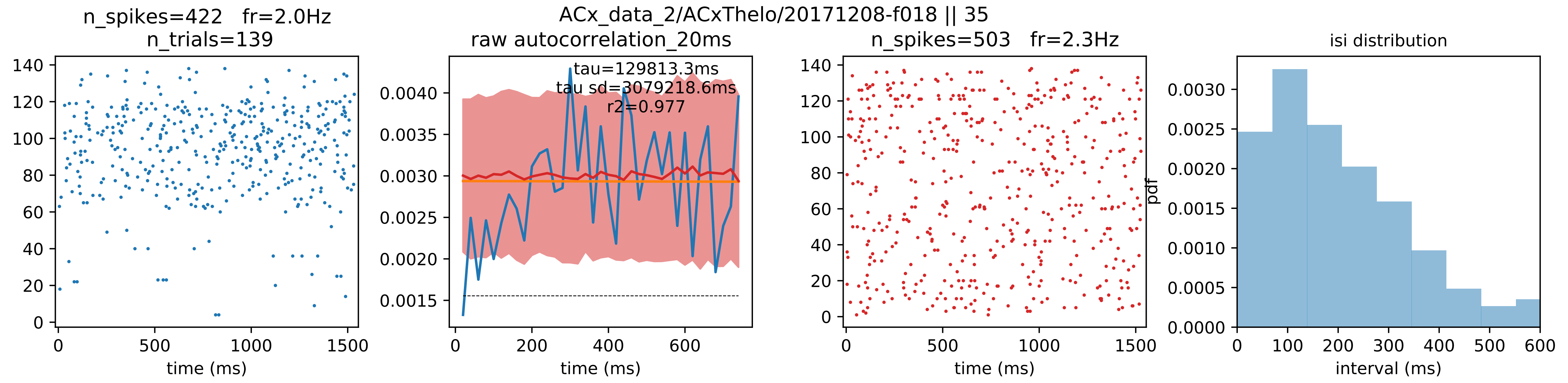

ACx_data_2/ACxThelo/20171208-f018 || 35
n_spikes=422 fr=2.0Hz
n_trials=139
time (ms)
raw autocorrelation_20ms
tau=129813.3ms
tau sd=3079218.6ms
r2=0.977
time (ms)
n_spikes=503 fr=2.3Hz
time (ms)
isi distribution
pdf
interval (ms)

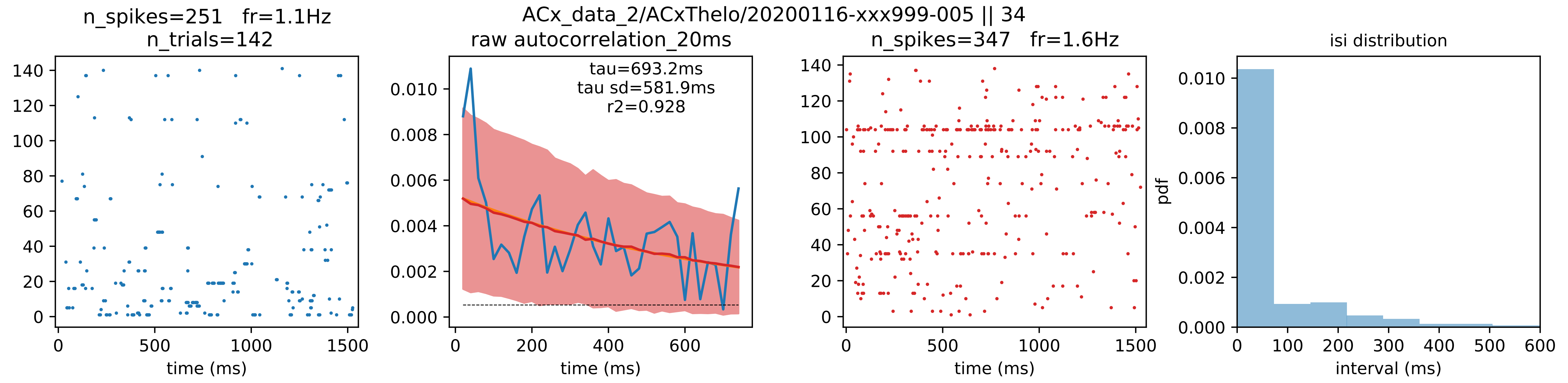
ACx_data_2/ACxThelo/20200116-xxx999-005 || 34
n_spikes=251 fr=1.1Hz
n_trials=142
time (ms)
raw autocorrelation_20ms
tau=693.2ms
tau sd=581.9ms
r2=0.928
time (ms)
n_spikes=347 fr=1.6Hz
time (ms)
isi distribution
pdf
interval (ms)

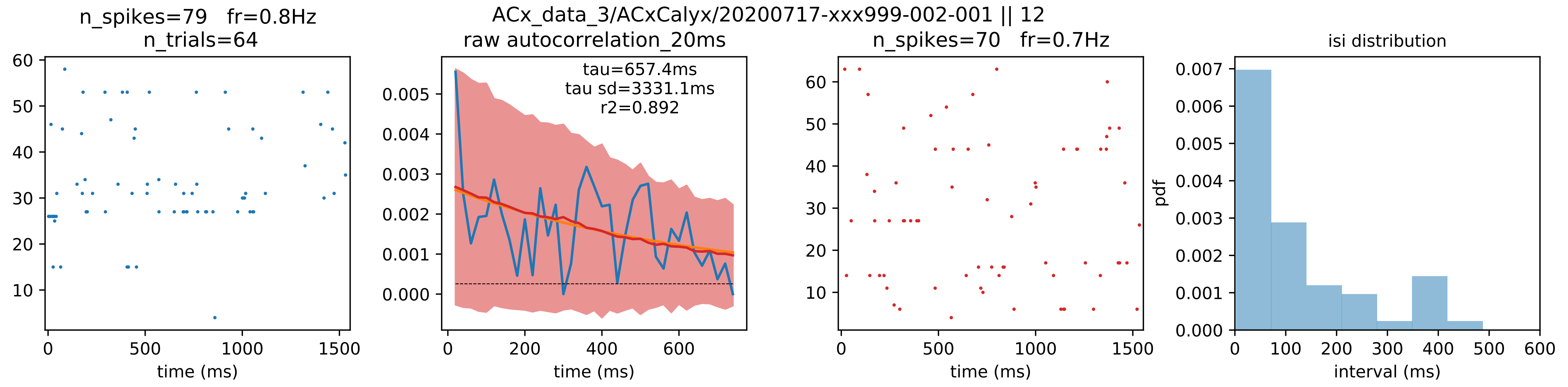

ACx_data_3/ACxCalyx/20200717-xxx999-002-001 || 12
n_spikes=79 fr=0.8Hz
n_trials=64
time (ms)
raw autocorrelation_20ms
tau=657.4ms
tau sd=3331.1ms
r2=0.892
time (ms)
n_spikes=70 fr=0.7Hz
time (ms)
isi distribution
pdf
interval (ms)

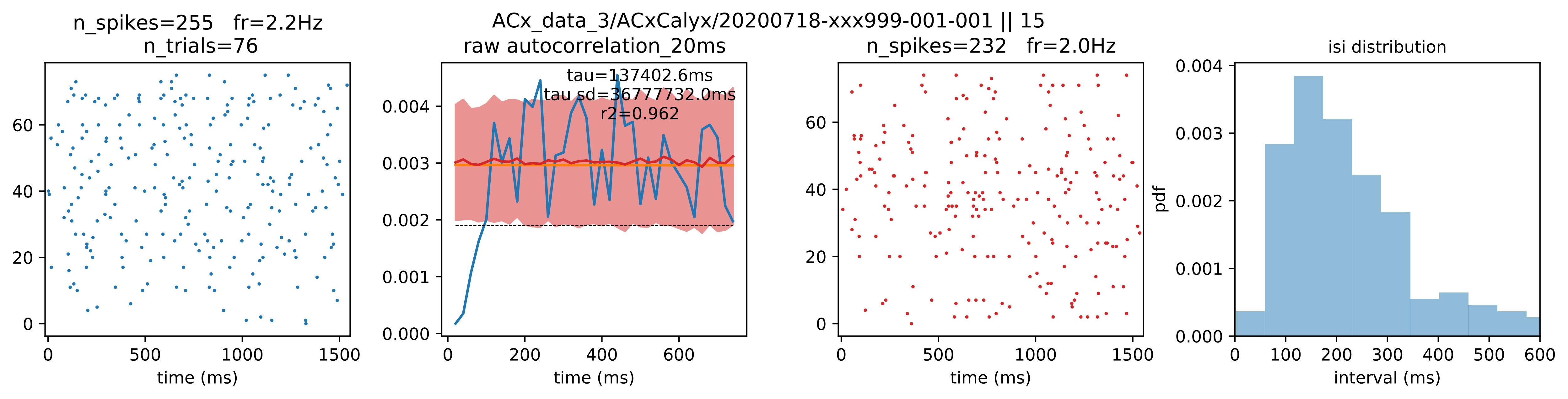
ACx_data_3/ACxCalyx/20200718-xxx999-001-001 || 15
n_spikes=255 fr=2.2Hz
n_trials=76
time (ms)
raw autocorrelation_20ms
tau=137402.6ms
tau sd=36777732.0ms
r2=0.962
time (ms)
n_spikes=232 fr=2.0Hz
time (ms)
isi distribution
pdf
interval (ms)

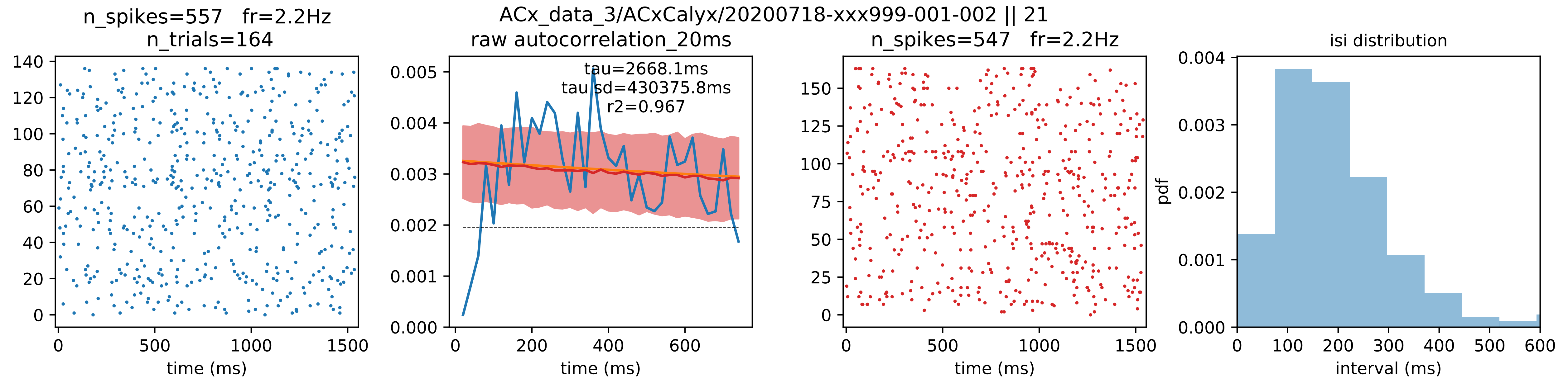
ACx_data_3/ACxCalyx/20200718-xxx999-001-002 || 21
n_spikes=557 fr=2.2Hz
n_trials=164
time (ms)
raw autocorrelation_20ms
tau=2668.1ms
tau sd=430375.8ms
r2=0.967
time (ms)
n_spikes=547 fr=2.2Hz
time (ms)
isi distribution
pdf
interval (ms)

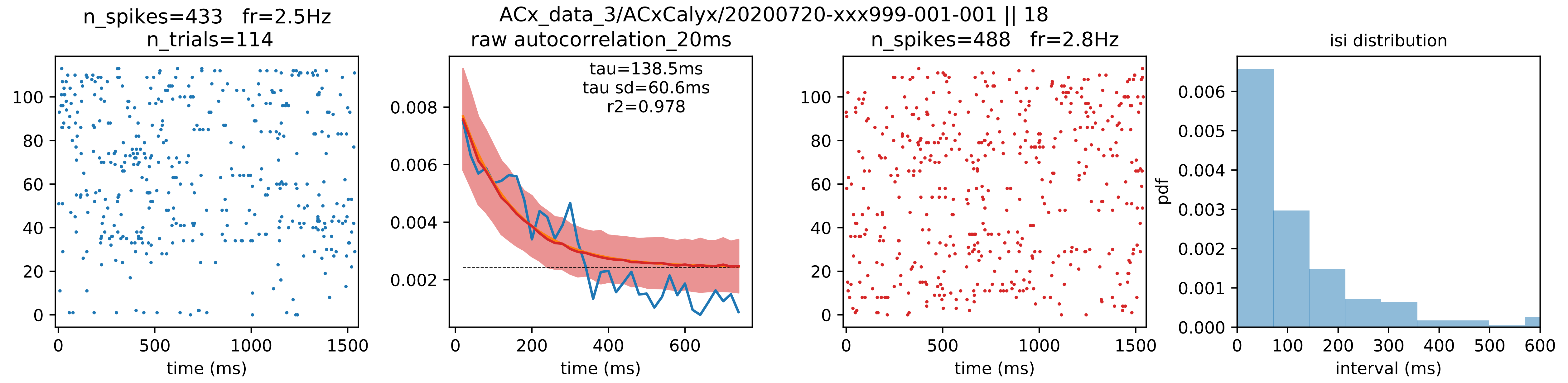
ACx_data_3/ACxCalyx/20200720-xxx999-001-001 || 18
n_spikes=433 fr=2.5Hz
n_trials=114
time (ms)
raw autocorrelation_20ms
tau=138.5ms
tau sd=60.6ms
r2=0.978
time (ms)
n_spikes=488 fr=2.8Hz
time (ms)
isi distribution
pdf
interval (ms)

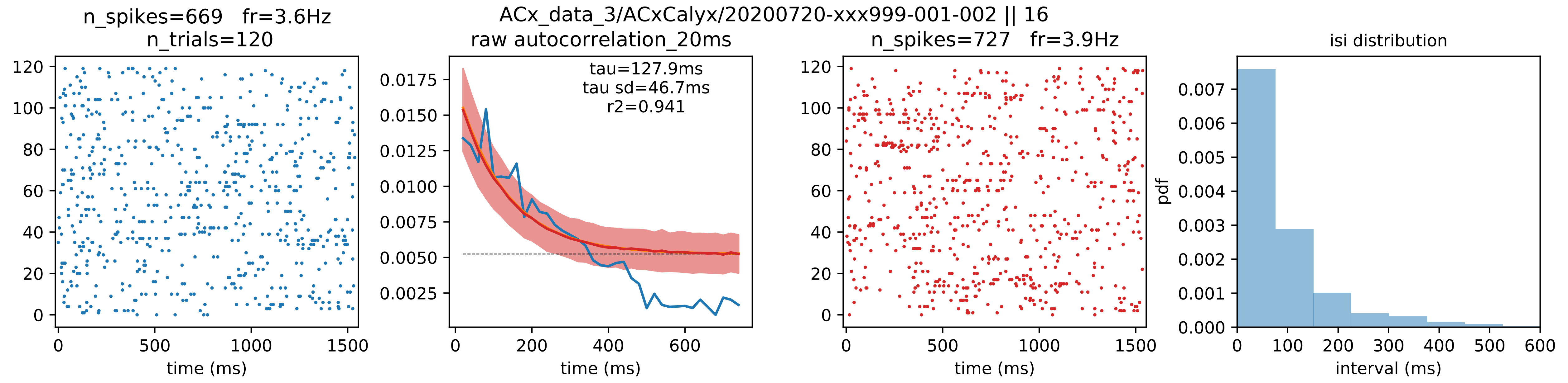

ACx_data_3/ACxCalyx/20200720-xxx999-001-002 || 16
n_spikes=669 fr=3.6Hz
n_trials=120
time (ms)
raw autocorrelation_20ms
tau=127.9ms
tau sd=46.7ms
r2=0.941
time (ms)
n_spikes=727 fr=3.9Hz
time (ms)
isi distribution
pdf
interval (ms)

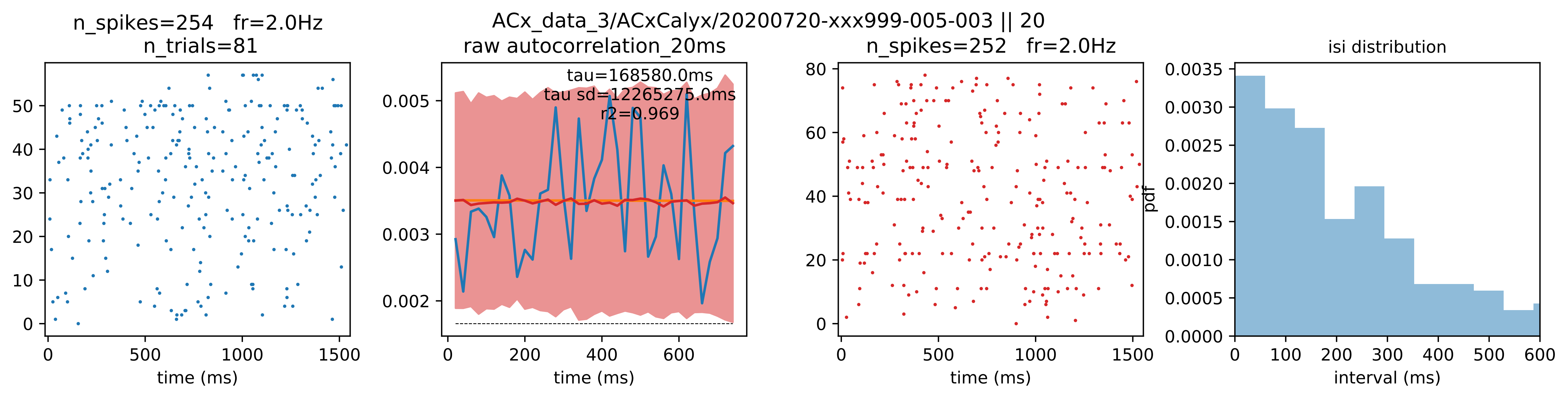
ACx_data_3/ACxCalyx/20200720-xxx999-005-003 || 20
n_spikes=254 fr=2.0Hz
n_trials=81
time (ms)
raw autocorrelation_20ms
tau=168580.0ms
tau sd=12265275.0ms
r2=0.969
time (ms)
n_spikes=252 fr=2.0Hz
time (ms)
isi distribution
pdf
interval (ms)

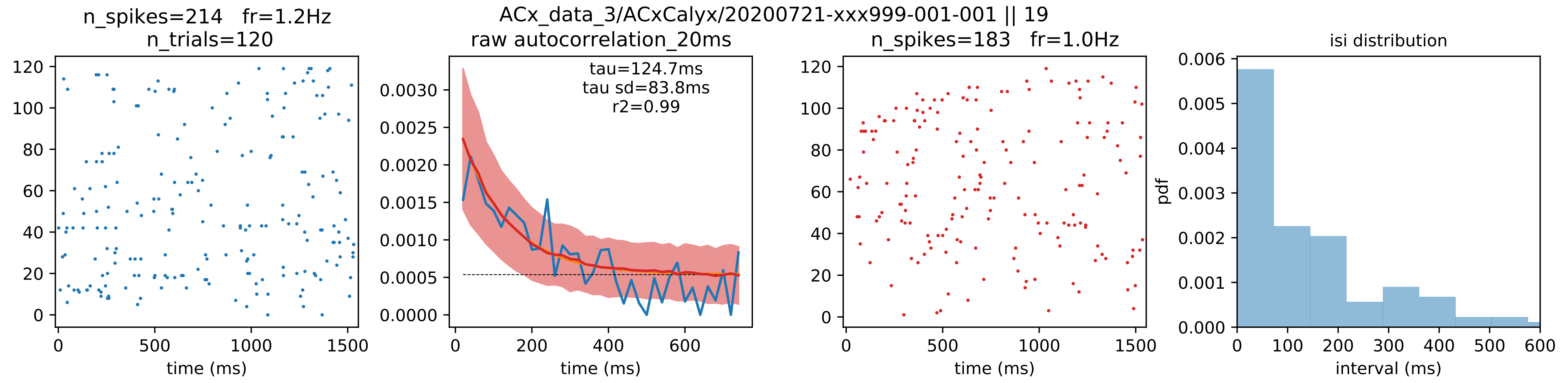

ACx_data_3/ACxCalyx/20200721-xxx999-001-001 || 19
n_spikes=214 fr=1.2Hz
n_trials=120
time (ms)
raw autocorrelation_20ms
tau=124.7ms
tau sd=83.8ms
r2=0.99
time (ms)
n_spikes=183 fr=1.0Hz
time (ms)
isi distribution
pdf
interval (ms)

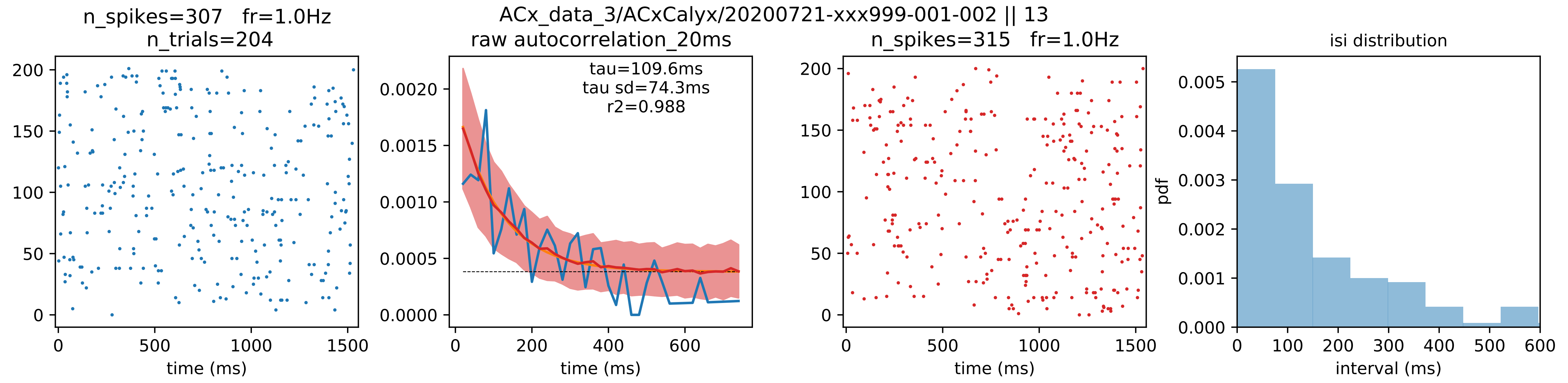

ACx_data_3/ACxCalyx/20200721-xxx999-001-002 || 13
n_spikes=307 fr=1.0Hz
n_trials=204
time (ms)
raw autocorrelation_20ms
tau=109.6ms
tau sd=74.3ms
r2=0.988
time (ms)
n_spikes=315 fr=1.0Hz
time (ms)
isi distribution
pdf
interval (ms)

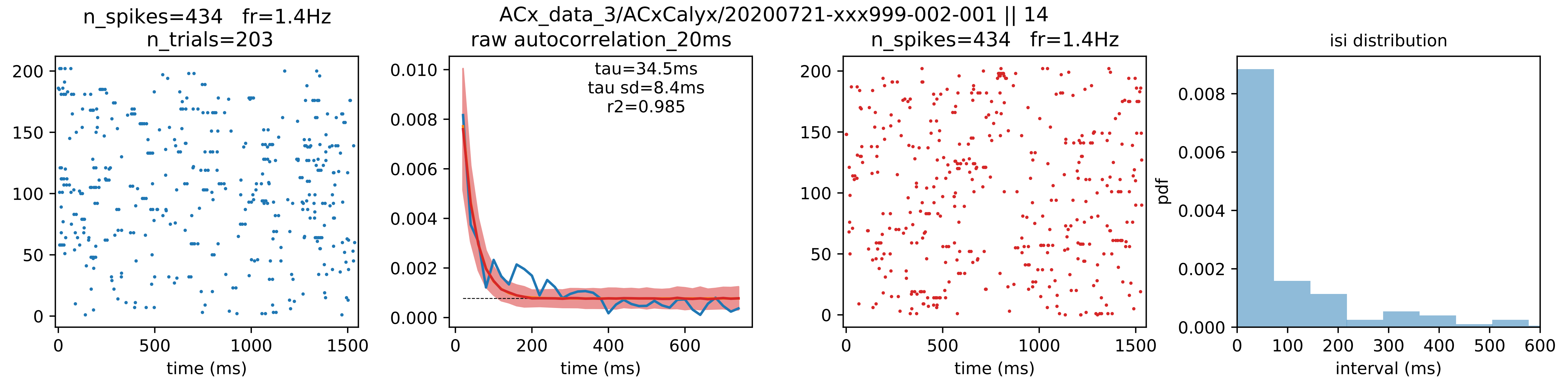
ACx_data_3/ACxCalyx/20200721-xxx999-002-001 || 14
n_spikes=434 fr=1.4Hz
n_trials=203
time (ms)
raw autocorrelation_20ms
tau=34.5ms
tau sd=8.4ms
r2=0.985
time (ms)
n_spikes=434 fr=1.4Hz
time (ms)
isi distribution
pdf
interval (ms)

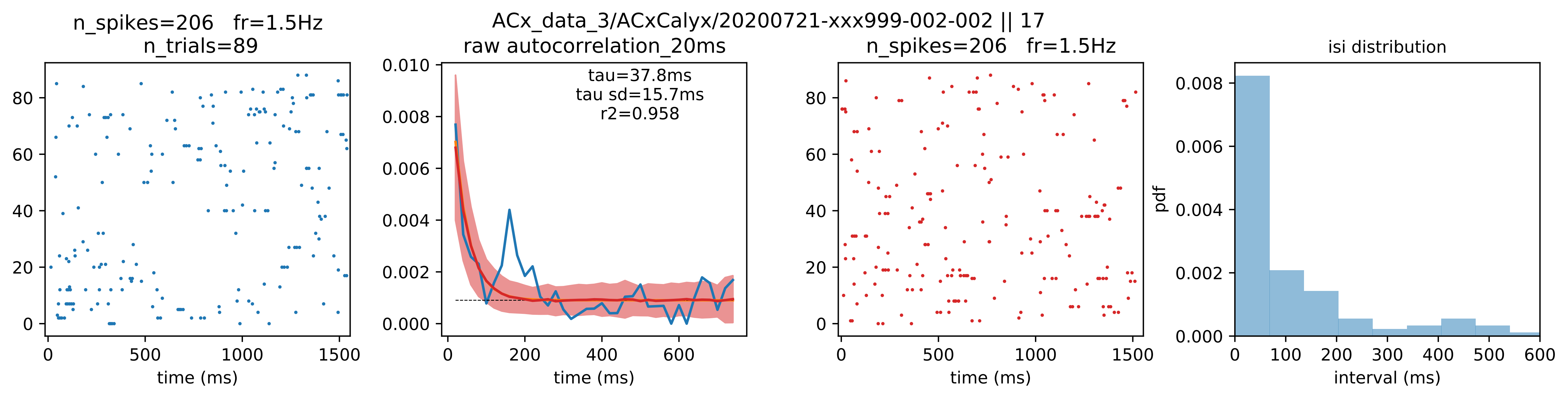

ACx_data_3/ACxCalyx/20200721-xxx999-002-002 || 17
n_spikes=206 fr=1.5Hz
n_trials=89
time (ms)
raw autocorrelation_20ms
tau=37.8ms
tau sd=15.7ms
r2=0.958
time (ms)
n_spikes=206 fr=1.5Hz
time (ms)
isi distribution
pdf
interval (ms)

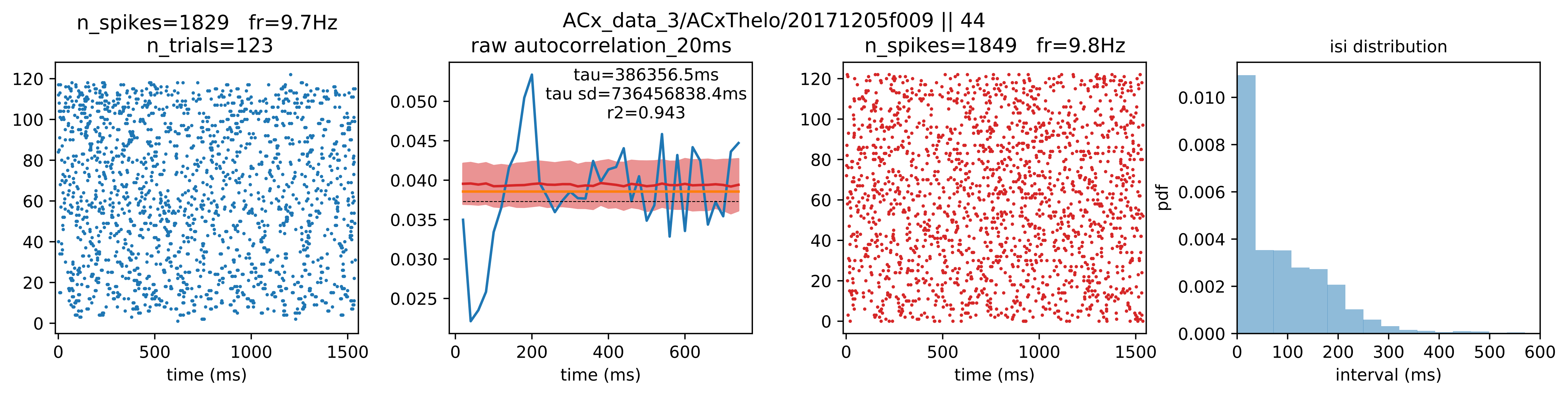

ACx_data_3/ACxThelo/20171205f009 || 44
n_spikes=1829 fr=9.7Hz
n_trials=123
time (ms)
raw autocorrelation_20ms
tau=386356.5ms
tau sd=736456838.4ms
r2=0.943
time (ms)
n_spikes=1849 fr=9.8Hz
time (ms)
isi distribution
pdf
interval (ms)

ACx_data_3/ACxThelo/20180219f012 || 43

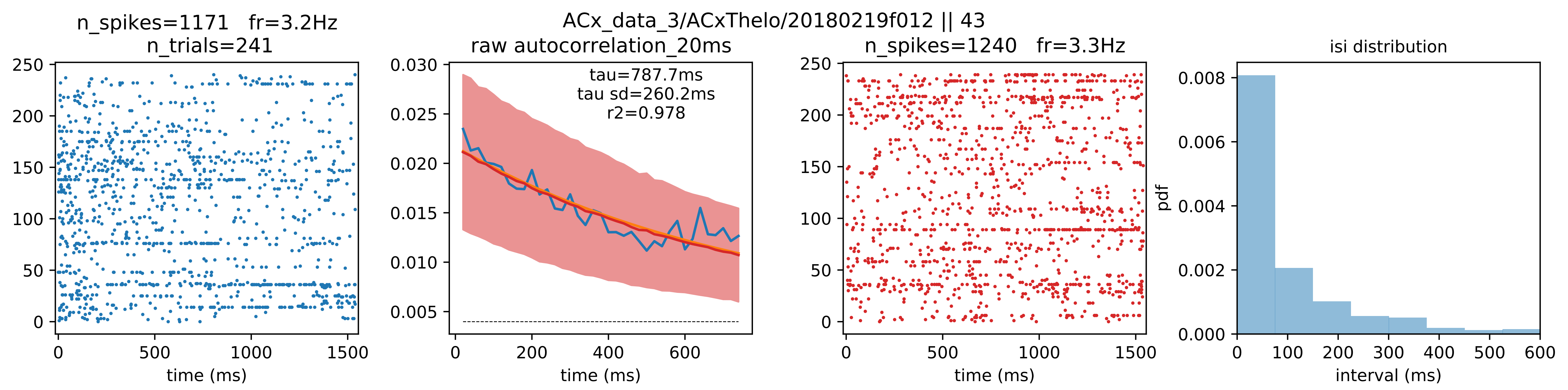

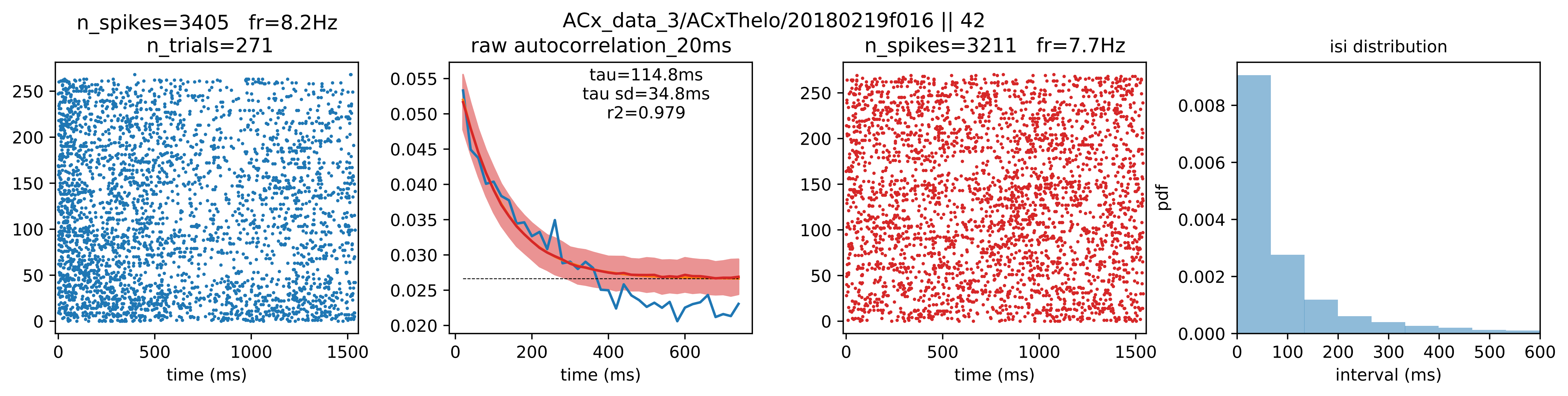

ACx_data_3/ACxThelo/20180219f016 || 42
n_spikes=3405 fr=8.2Hz
n_trials=271
time (ms)
raw autocorrelation_20ms
tau=114.8ms
tau sd=34.8ms
r2=0.979
time (ms)
n_spikes=3211 fr=7.7Hz
time (ms)
isi distribution
pdf
interval (ms)

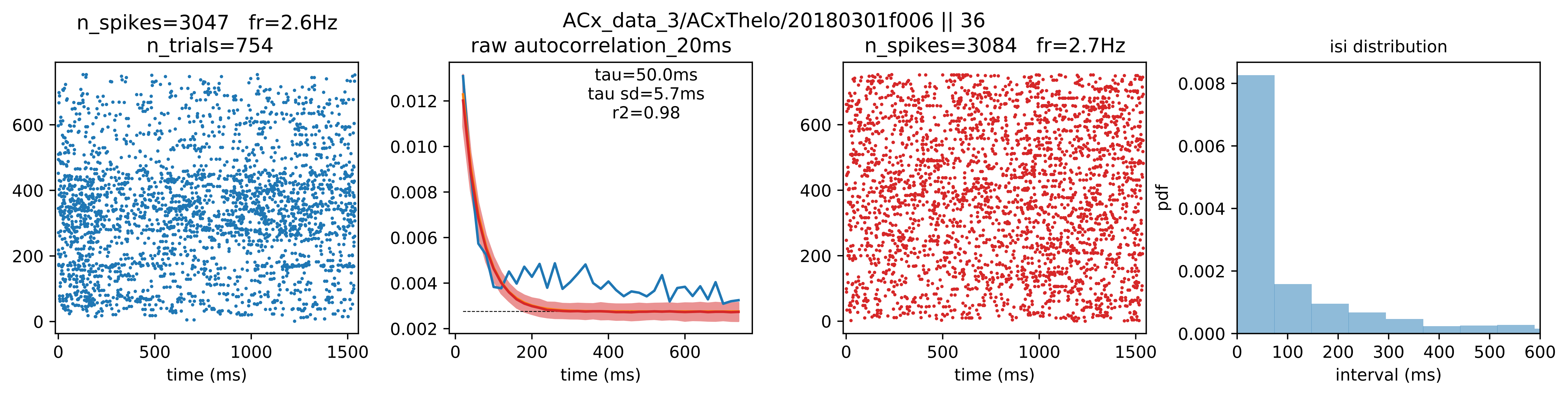

ACx_data_3/ACxThelo/20180301f006 || 36
n_spikes=3047 fr=2.6Hz
n_trials=754
time (ms)
raw autocorrelation_20ms
tau=50.0ms
tau sd=5.7ms
r2=0.98
time (ms)
n_spikes=3084 fr=2.7Hz
time (ms)
isi distribution
pdf
interval (ms)

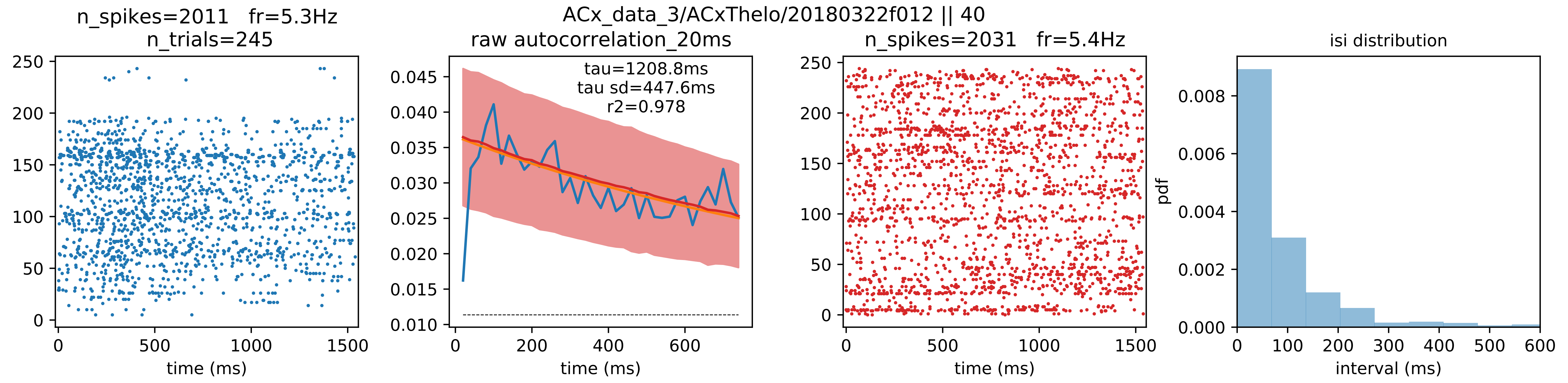
ACx_data_3/ACxThelo/20180322f012 || 40
n_spikes=2011 fr=5.3Hz
n_trials=245
time (ms)
raw autocorrelation_20ms
tau=1208.8ms
tau sd=447.6ms
r2=0.978
time (ms)
n_spikes=2031 fr=5.4Hz
time (ms)
isi distribution
pdf
interval (ms)

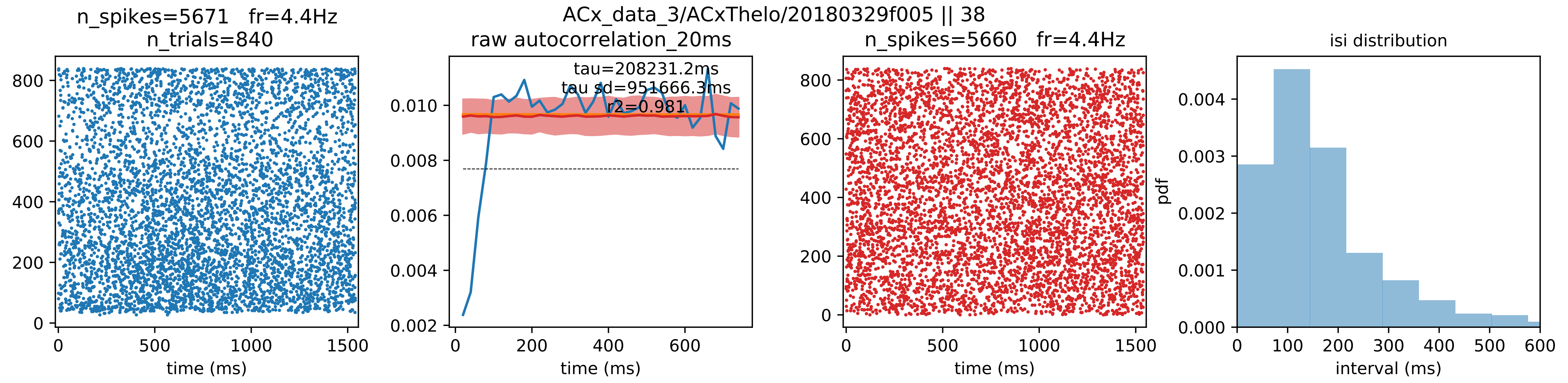

ACx_data_3/ACxThelo/20180329f005 || 38
n_spikes=5671 fr=4.4Hz
n_trials=840
time (ms)
raw autocorrelation_20ms
tau=208231.2ms
tau sd=951666.3ms
r2=0.981
time (ms)
n_spikes=5660 fr=4.4Hz
time (ms)
isi distribution
pdf
interval (ms)

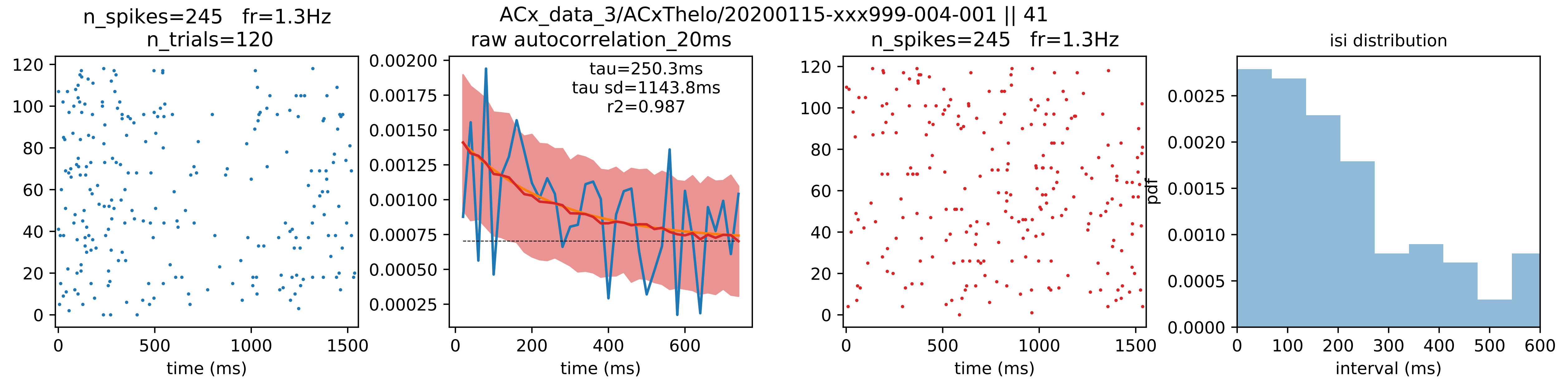
ACx_data_3/ACxThelo/20200115-xxx999-004-001 || 41
n_spikes=245 fr=1.3Hz
n_trials=120
time (ms)
raw autocorrelation_20ms
tau=250.3ms
tau sd=1143.8ms
r2=0.987
time (ms)
n_spikes=245 fr=1.3Hz
time (ms)
isi distribution
pdf
interval (ms)

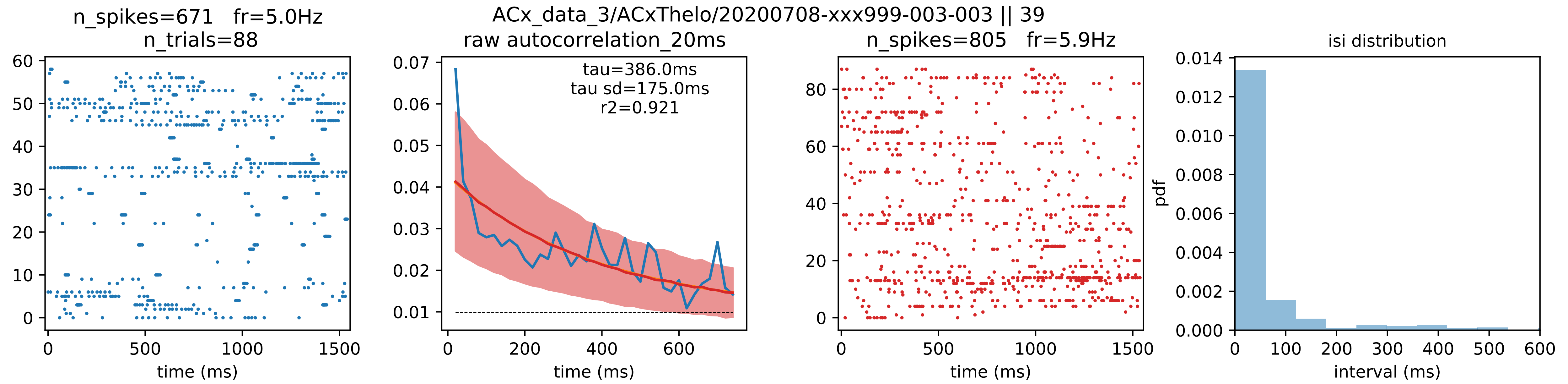
ACx_data_3/ACxThelo/20200708-xxx999-003-003 || 39
n_spikes=671 fr=5.0Hz
n_trials=88
time (ms)
raw autocorrelation_20ms
tau=386.0ms
tau sd=175.0ms
r2=0.921
time (ms)
n_spikes=805 fr=5.9Hz
time (ms)
isi distribution
pdf
interval (ms)

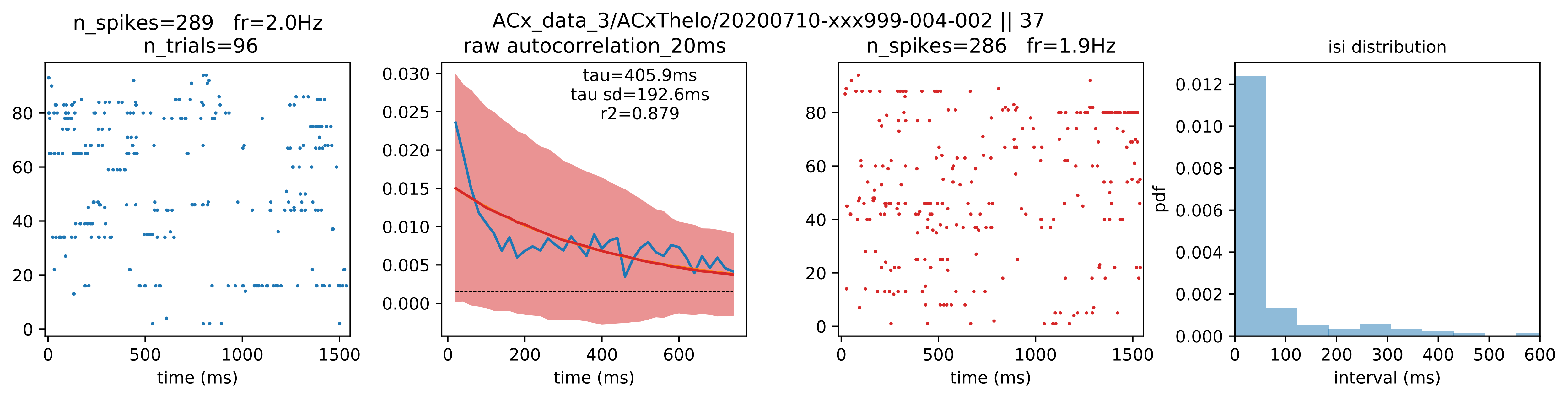

n_spikes=289 fr=2.0Hz
n_trials=96
ACx_data_3/ACxThelo/20200710-xxx999-004-002 || 37
raw autocorrelation_20ms
tau=405.9ms
tau sd=192.6ms
r2=0.879
n_spikes=286 fr=1.9Hz
isi distribution
time (ms)
interval (ms)
pdf
